# The MRN complex maintains the biliary-derived hepatocytes in liver regeneration through ATR-Chk1 pathway

**DOI:** 10.1038/s41536-023-00294-3

**Published:** 2023-04-06

**Authors:** Jingmei Song, Jianlong Ma, Xing Liu, Zhuofu Huang, Lianghui Li, Linke Li, Lingfei Luo, Rui Ni, Jianbo He

**Affiliations:** Institute of Developmental Biology and Regenerative Medicine, Southwest University, Beibei, Chongqing, China

**Keywords:** Liver diseases, Regeneration, Cell death

## Abstract

When the proliferation of residual hepatocytes is prohibited, biliary epithelial cells (BECs) transdifferentiate into nascent hepatocytes to accomplish liver regeneration. Despite significant interest in transdifferentiation, little is known about the maintenance of nascent hepatocytes in post-injured environments. Here, we perform an N-ethyl-N-nitrosourea (ENU) forward genetic screen and identify a mutant containing a nonsense mutation in the gene *nibrin* (*nbn*), which encodes a component of the Mre11-Rad50-Nbn (MRN) complex that activates DNA damage response (DDR). The regenerated hepatocytes cannot be maintained and exhibit apoptosis in the mutant. Mechanistically, the *nbn* mutation results in the abrogation of ATR-Chk1 signaling and accumulations of DNA damage in nascent hepatocytes, which eventually induces p53-mediated apoptosis. Furthermore, loss of *rad50* or *mre11a* shows similar phenotypes. This study reveals that the activation of DDR by the MRN complex is essential for the survival of BEC-derived hepatocytes, addressing how to maintain nascent hepatocytes in the post-injured environments.

## Introduction

The liver is a vital organ with a unique regenerative capacity. Upon varying degrees of injury, the regenerating liver parenchymal cells are derived from pre-existing hepatocytes or biliary epithelial cells (BECs)^1–5^. Many studies have reported that ductular reactions occur in certain pathological conditions such as viral hepatitis, alcoholic hepatitis, nonalcoholic steatohepatitis, and liver cirrhosis^6,7^. Recent findings further show that BECs extensively contribute to regenerated hepatocytes upon severe liver injury in zebrafish^8,9^ and mice^10–13^ when hepatocyte-mediated regeneration is compromised. BECs dedifferentiate into bi-potential progenitor cells (BPPCs) and subsequently redifferentiate and proliferate into hepatocytes and BECs in zebrafish^8,9^. Considering the crucial roles of hepatocytes in physiological functions, it is necessary to explore the mechanisms underlying the formation and survival of regenerated hepatocytes for the clinical therapeutics of liver diseases.

Liver regeneration is a complex and highly regulated process that ensures damaged hepatic structural and functional restoration. When hepatocytes are severely injured, or the proliferative capacity of residual hepatocytes is inhibited in zebrafish or mouse models^8–13^, nascent hepatocytes mainly originate from BECs. Given the conservation between both species, understanding the mechanisms underlying the generation and maintenance of regenerated hepatocytes will be useful for elucidating the regulatory mechanisms underlying liver regeneration in end-stage liver diseases. Recently, our group addressed that the mammalian target of rapamycin complex 1 signaling and DNA methyltransferase 1 are required for the BEC-to-BPPC dedifferentiation after extreme liver damage^14,15^. Other studies have suggested farnesoid X receptor-extracellular signal-related kinase 1 axis, Wnt, and bone morphogenetic protein govern the BPPC-to-hepatocyte redifferentiation^16–18^, and Notch regulates the BPPC-to-BEC redifferentiation^19^. Although many factors involved in cell transdifferentiation have been identified, the molecular mechanisms underlying the maintenance of regenerated hepatocytes remain largely unknown.

The MRN complex, composed of Mre11, Rad50, and Nbn, is involved in the maintenance of genomic stability, DNA repair, and cell cycle regulation. Besides, the MRN complex is an extensively documented sensor of DNA damage response (DDR) that detects DNA double-strand breaks (DSBs) and replication stress to fully activate both ataxia telangiectasia mutated (ATM) and RAD3-related (ATR)-dependent signalings^20,21^. ATM and ATR are the key mediators of the DDR. Upon recruitment to the sites of DNA damage, ATM and ATR phosphorylate downstream substrates Chk2 and Chk1, respectively, to induce cell cycle arrest and initiate DNA repair^22–25^. Subsequently, p53 is activated to allow repair of DNA damage or undergo apoptosis if DNA damage is irreparable. Although the MRN complex plays an important role in DDR, its function in liver regeneration, particularly in extreme hepatocyte injury, has not been addressed.

For the first time, our group undertook an N-ethyl-N-nitrosourea (ENU)-mutagenesis-based forward genetic screen of liver regeneration in zebrafish to explore the mechanisms underlying liver regeneration after extreme hepatocyte loss. We identified a mutation in *nibrin* (*nbn*) by positional cloning and reverse genetics approaches, which shows severe defects during the later stage of liver regeneration, but does not affect the transdifferentiation process. Further, we used *rad50* mutant and chemical inhibition of ATM, ATR, or Chk1 to reveal MRN-ATR-Chk1 axis is indispensable for the survival of regenerated hepatocytes. Besides, cell apoptosis induced by impaired MRN complex is p53-dependent during liver regeneration in vivo. In summary, this study suggests the essential role of MRN complex in maintaining the survival of hepatocytes during BECs-driven liver regeneration.

## Results

### The zebrafish *lvv*^*cq137*^ mutant displays defective maintenance of nascent hepatocytes in liver regeneration after extreme liver injury

**Fig. 1:**
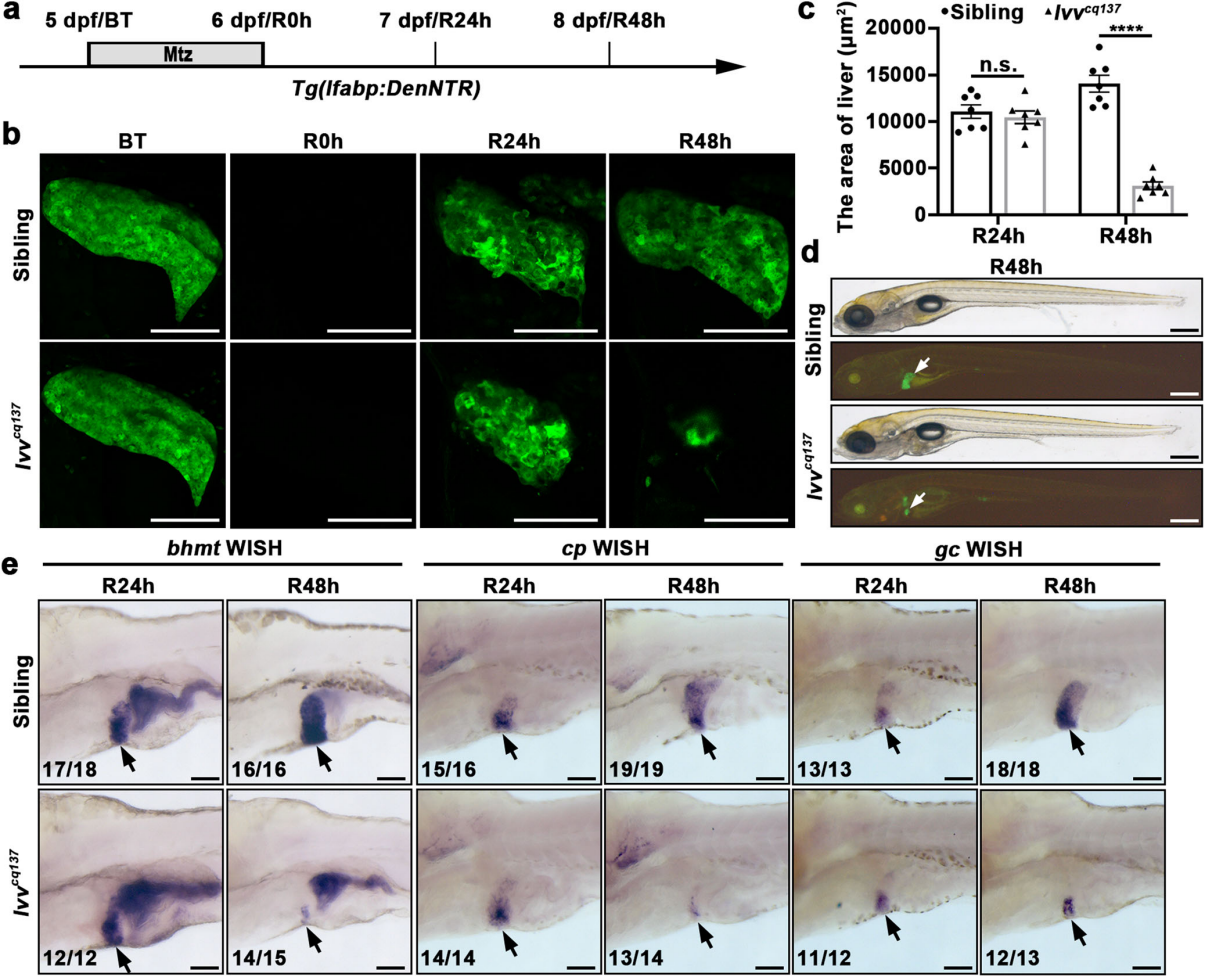
The maintenance of nascent hepatocytes is defective in *lvv* mutant in liver regeneration. **a** Schematic illustration of Mtz treatment and analysis at BT (5 dpf), R0h (6 dpf), R24h (7 dpf), and R48h (8 dpf). **b** Confocal projection images showing the regenerating livers of sibling and *lvv* mutant from BT to R48h under the *Tg(lfabp:DenNTR)* background. **c** Quantification of the area of liver sizes, *n* = 7 larvae per group. **d** Bright-field and epifluorescence images showing the morphological appearance and regenerating livers (arrows) of sibling and *lvv* larvae at R48h, respectively. **e** WISH images showing the expressions of *bhmt*, *cp*, and *gc* in regenerating livers (arrows) at R24h and R48h in sibling and *lvv* larvae. Asterisks indicate statistical significance: ****P < 0.0001 using *t*-tests analysis when compared to control. Numbers indicate the proportion of larvae exhibiting the phenotype shown. Scale bars, 100 μm **b**, **e**, and 300 μm **d**; error bars, ±SEM. BT before Mtz treatment, R regeneration time after the withdrawal of Mtz, WISH whole-mount in situ hybridization, n.s. no significance.

To explore novel genes and regulatory mechanisms in liver regeneration, we carried out an N-ethyl-N-nitrosourea (ENU) forward genetics screen under the *Tg(lfabp:DenNTR)*^*cq1*^ background^8^, in which liver fatty acid-binding protein 10a (*lfabp*) promoter drives the expression of Dendra2 and nitroreductase (NTR) fusion protein in hepatocytes. The *Tg(lfabp:DenNTR)* transgenic larvae were treated with 10 mmol/L metronidazole (Mtz) for 24 hours from 5 days post-fertilization (dpf) (Fig. 1a). Then, hepatocyte regeneration could be prominently observed at regeneration 24 hours (R24h) after withdrawal of Mtz. The regenerating liver restored 50% of its normal size at R48h and fully regenerated within the next two days^8,9^. After the screen, we identified a recessive mutant named liver victor *(lvv)*^*cq137*^, which exhibited relatively normal liver size at R24h but dramatically reduced at R48h in contrast to the siblings (Fig. 1b, c). Overall, no obvious developmental defects were observed in the *lvv* mutant (Fig. 1d). To confirm the defect of liver regeneration in *lvv* mutant, we further examined the expressions of hepatic markers, such as betaine-homocysteine methyltransferase (*bhmt*), ceruloplasmin (*cp*), and vitamin D binding protein (*gc*) by whole-mount in situ hybridization (WISH). The expression intensity and area of *bhmt*, *cp*, and *gc* were comparable between mutants and siblings at R24h (Fig. 1e). In contrast, the expression area of these markers substantially decreased in the mutants at R48h (Fig. 1e). These results suggest the maintenance of nascent hepatocytes is defective during liver regeneration in *lvv* mutant.

To exclude the developmental defect of BECs and bile secretion in *lvv* mutant, we performed a feeding assay with the fluorescent BODIPY FL C5, a fatty acid analogue that helps to visualize the bile secretion of hepatocytes and the bile conduction in bile ducts^26^. Normal accumulation of BODIPY FL C5 in the bile ducts and gallbladder at 5 dpf indicated that hepatocytes and BECs are functional in *lvv* mutant (Supplementary Fig. 1a). We also examined the morphology of bile ducts using the *Tg(Tp1:Tomato)* line^15,16^, and found the biliary duct system was well developed in *lvv* mutant (Supplementary Fig. 1b). To confirm whether BEC transdifferentiation was disturbed in *lvv* mutant, the Cre/loxP-mediated lineage tracing was performed using the *Tg(krt18:CreER)* transgenic line^14,15^. Validated by the *Tg(krt18:CreER; βactin:loxP-DsRed-loxP-GFP)* (Supplementary Fig. 2a, b), almost all the nascent hepatocytes derived from the transdifferentiation of BECs in the *Tg(krt18:CreER; lfabp:loxP-STOP-loxP-DsRed; lfabp:DenNTR)* line in sibling and *lvv* mutant (Supplementary Fig. 2c, d). These data suggest that the origins of nascent hepatocytes are unaffected in *lvv* mutant.

As the mutant exhibited normal liver sizes from 5 to 8 days post-fertilization (dpf) (Supplementary Fig. 3a), we further checked the development of digestive organs using ceruloplasmin (*cp*), prospero homeobox 1a (*prox1a*), fatty acid binding protein 2 (*fabp2*), *trypsin*, and *insulin* probes. WISH results showed that the liver, intestine, and pancreas develop normally in *lvv* mutant (Supplementary Fig. 3b, c). These results indicate that the *lvv* mutant explicitly affects liver regeneration without obvious developmental defects.

### Liver regeneration defect in *lvv* mutant is caused by the mutation of *nbn*

**Fig. 2:**
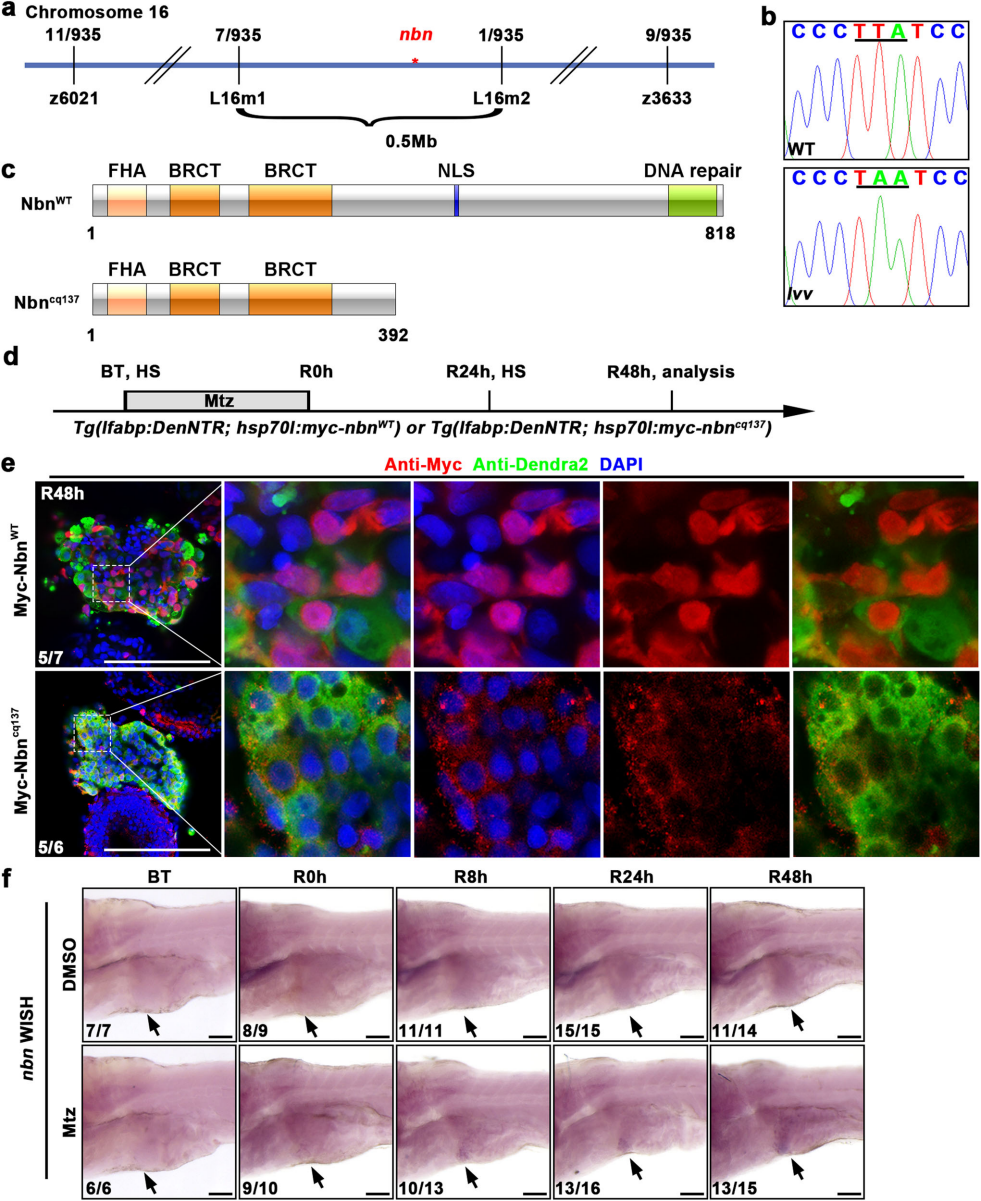
*nbn* is the mutated gene of *lvv* mutant. **a** Genetic map of the candidate region on chromosome 16. Numbers above SSLP markers indicate recombination events. **b** Sequencing results displaying the mutation site in *nbn*. **c** Schematic diagram of the structure of Nbn protein. FHA, BRCT, and NLS denote forkhead-associated domain, BRCA1 C-terminal domain, and nuclear localization signal, respectively. **d** Schematic illustration of Mtz treatment and analysis at R48h. **e** Single optical section images showing Myc (red), Dendra2 (green), and DAPI (blue) staining in regenerating livers at R48h under the *Tg(lfabp:DenNTR)* background. Note that Myc-tagged Nbn^WT^ is mainly localized in the nucleus, while Myc-tagged Nbn^cq137^ is localized in the cytoplasm. **f** WISH images showing *nbn* expression in the liver region (arrows) from BT to R48h. Numbers indicate the proportion of larvae exhibiting the expression shown. Scale bars, 100 μm. WT wild-type, DAPI 4′, 6-diamidino-2-phenylindole, BT before Mtz treatment, R regeneration time after the withdrawal of Mtz, WISH whole-mount in situ hybridization.

To identify the target gene of *lvv*, we used the positional cloning approach to map the mutation site. The mutation was localized within seven candidate genes flanked by two closely linked simple sequence length polymorphism (SSLP) markers, L16m1 and L16m2, on chromosome 16 (Fig. 2a). After sequencing with cDNA and genomic DNA in *lvv* and sibling, we identified a candidate mutation in the *nbn* gene within this linked region. The T to A nonsense mutation was identified (Leu to STOP coding transition) and predicted to truncate Nbn prior to the putative nuclear localization signal (NLS) and DNA repair domain; thus, the mutation is predicted to be a loss-of-function allele (Fig. 2b, c). Then we analyzed the subcellular localization of Nbn^cq137^. A Myc-tag was fused to the N-terminus of wild-type Nbn (Nbn^WT^) protein and mutated Nbn (Nbn^cq137^) protein for detection, respectively (Fig. 2d). Confocal images showed that Myc-tagged Nbn^WT^ was predominantly localized in the nucleus of regenerated hepatocytes (Fig. 2e). However, Myc-tagged Nbn^cq137^ was mistakenly localized in the cytoplasm (Fig. 2e), which is consistent with the lack of putative NLS in Nbn^cq137^. These results suggest that the Nbn^cq137^ cannot form the nuclear foci and loses its nuclear function of DNA repair. It has been reported that Nbn is mainly expressed in regions of high proliferative activity or organs with physiologic DSBs^27^. We examined the spatiotemporal pattern of *nbn* expression. WISH assay showed the expression of *nbn* mRNA was gradually upregulated from R8h to R48h in the liver region during liver regeneration (Fig. 2f), which indicates *nbn* plays a role in the later stage of liver regeneration.

To confirm the disruption of *nbn* was causative for the phenotype of *lvv* mutant, we generated a new *nbn* mutant line *nbn*^*cq139*^ using the CRISPR/Cas9 system. Due to the ENU nonsense mutation causing premature termination of the Nbn protein, we designed two specific gRNAs based on the sequences of *nbn* in exon 11 and exon 15 for large fragment deletion to mimic the ENU mutation (Supplementary Fig. 4a). Wild-type alleles were too long to be amplified under conventional PCR conditions, while the knockout produces a 500 bp fragment that can be separated by gel electrophoresis (Supplementary Fig. 4b). The *nbn*^*cq139*^ mutant phenocopied the liver regeneration defects of *lvv*; a relatively normal liver size was formed at R24h, and the liver size decreased dramatically at R48h (Supplementary Fig. 4c, d). To further confirm whether the disrupted *nbn* was responsible for the defective liver regeneration, *Tg(hsp70l:nbn-p2A-mCherry)*^*cq141*^ was generated for overexpression of Nbn in the *lvv* mutant after heat-shock induction (Supplementary Fig. 4e). Liver regeneration defect in *lvv* mutant could be rescued by Nbn overexpression, which was identified by the red fluorescence of mCherry (Supplementary Fig. 4e, f). These results validate that the phenotype of impaired liver regeneration in *lvv* mutant is caused by *nbn* mutation.

### *Nbn* maintains liver regeneration depending on the function of MRN complex

**Fig. 3:**
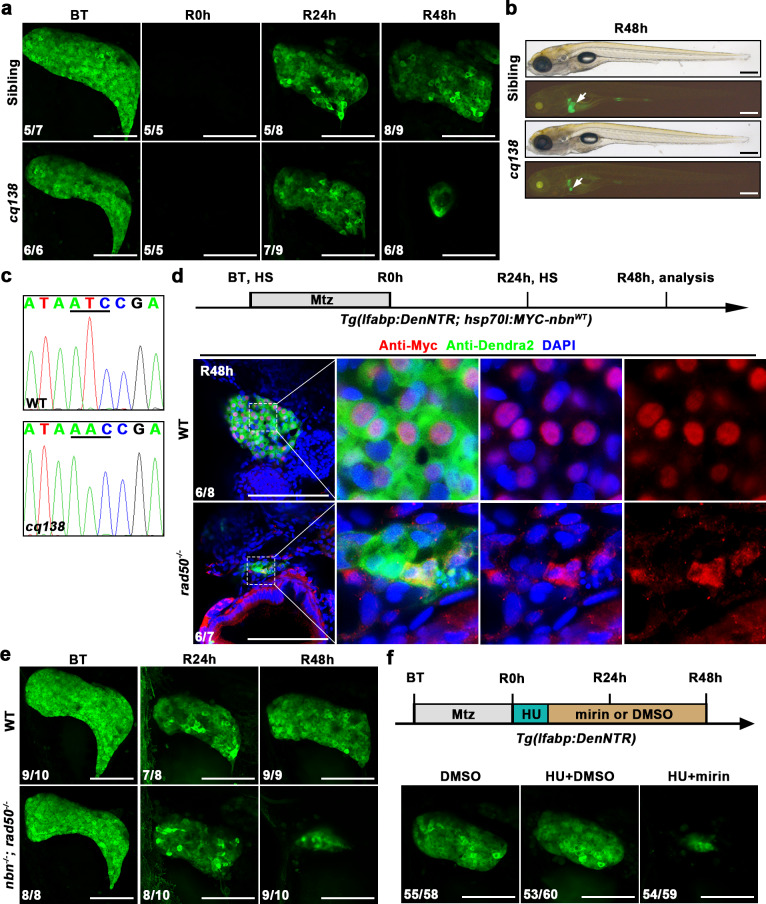
The components of MRN complex are required for liver regeneration. **a** Confocal projection images showing the regenerating livers of sibling and *cq138* mutant from BT to R48h under the *Tg(lfabp:DenNTR)* background. **b** Bright-field and epifluorescence images showing the morphological appearance and regenerating livers (arrows) of sibling and *cq138* larvae at R48h, respectively. **c** Sequencing result displaying the mutation site in *rad50*. **d** Schematic illustration of Mtz treatment and analysis at R48h. Single optical section images showing Myc (red), Dendra2 (green), and DAPI (blue) staining in regenerating livers of wild-type and *cq138* mutant at R48h under the *Tg(lfabp:DenNTR)* background. Note that Myc-tagged Nbn^WT^ is mislocalized to the cytoplasm in *rad50*^*-/-*^ larvae. **e** Confocal projection images showing the regenerating livers of wild-type and *nbn*^*-/-*^ *rad50*^*-/-*^ double mutant from BT to R48h under the *Tg(lfabp:DenNTR)* background. **f** Schematic illustration of HU and mirin treatment and analysis at R48h. Confocal projection images showing the regenerating livers of larvae treated with or without mirin under HU exposure. Numbers indicate the proportion of larvae exhibiting the expression shown. Scale bars, 100 μm (**a**, **d**, **e**, and **f**) and 300 μm **b**. BT before Mtz treatment, R regeneration time after the withdrawal of Mtz, WT wild-type, DAPI 4′, 6-diamidino-2-phenylindole, HU hydroxyurea.

Coincidentally, we identified another ENU-induced mutant *cq138* that showed dramatic liver size reduction at R48h in contrast to the sibling, while the liver size had no significant difference between siblings and *cq138* mutant at R24h (Fig. 3a). In general appearance, no obvious developmental defects were observed in the *cq138* mutant (Fig. 3b). In addition, the development of digestive organs such as the liver, intestine, and pancreas in *cq138* mutant were normal (Supplementary Fig. 5). These results indicate that the *cq138* mutant does not cause obvious developmental defects but specifically affects the process of the later stage of liver regeneration, which phenocopies the *nbn* mutant.

We identified *rad50*, another component of the MRN complex, as the mutation gene of *cq138* by positional cloning and sequencing. A point mutation from T to A replacement in the exon 25 of the *rad50* resulted in an Ile to Asn conversion (Fig. 3c). To further determine the identity of the mutated gene in *cq138*, we generated another mutant of *cq140* that carried a 6 bp deletion and 7 bp insertion by targeting the exon 15 of *rad50* using CRISPR/Cas9 technology (Supplementary Fig. 6a, b). *Rad50*^*cq140*^ could well recapitulate the phenotype of ENU-induced *rad50*^*cq138*^ (Supplementary Fig. 6c, d).

Rad50, together with Nbn and Mre11, forms an evolutionary conserved MRN complex in vertebrates^28–30^. To examine whether *nbn* and *rad50* act in a complex manner during liver regeneration, we analyzed the subcellular localization of Myc-tagged Nbn^WT^ in *rad50* mutant. Confocal imaging showed that Myc-Nbn^WT^ was mislocalized in the cytoplasm in *rad50* mutant (Fig. 3d). Since Myc-Nbn^cq137^ was cytoplasmic in wild-type (Fig. 2e) and Myc-Nbn^WT^ could not form nuclear foci in *rad50* mutant (Fig. 3d), we inferred that recruitment of the MRN complex to the DNA damage site was disturbed in both *nbn* and *rad50* mutant larvae. Next, we intercrossed *nbn* and *rad50* heterozygotes to generate a double mutant. The *nbn*^*-/-*^ *rad50*^*-/-*^ double mutant exhibited identical liver regeneration defects as *nbn* or *rad50* mutant (Fig. 3e), indicating that the effects of *nbn* and *rad50* were not additive. Furthermore, we observed that the addition of mirin, a characterized inhibitor of Mre11 nuclease activity, to hydroxyurea (HU) exposure, which could trigger replication fork stalling and induce DNA damage^31,32^, showing a phenotype similar to *nbn* mutation and *rad50* mutation (Fig. 3f). All three components of the MRN complex were indispensable for liver regeneration. These results suggest that *nbn*, *rad50*, and *mre11a* work as a complex in liver regeneration, and the integrity of the MRN complex is required to maintain nascent hepatocytes in liver regeneration.

### Loss of *nbn* induces cell apoptosis of nascent hepatocytes

**Fig. 4:**
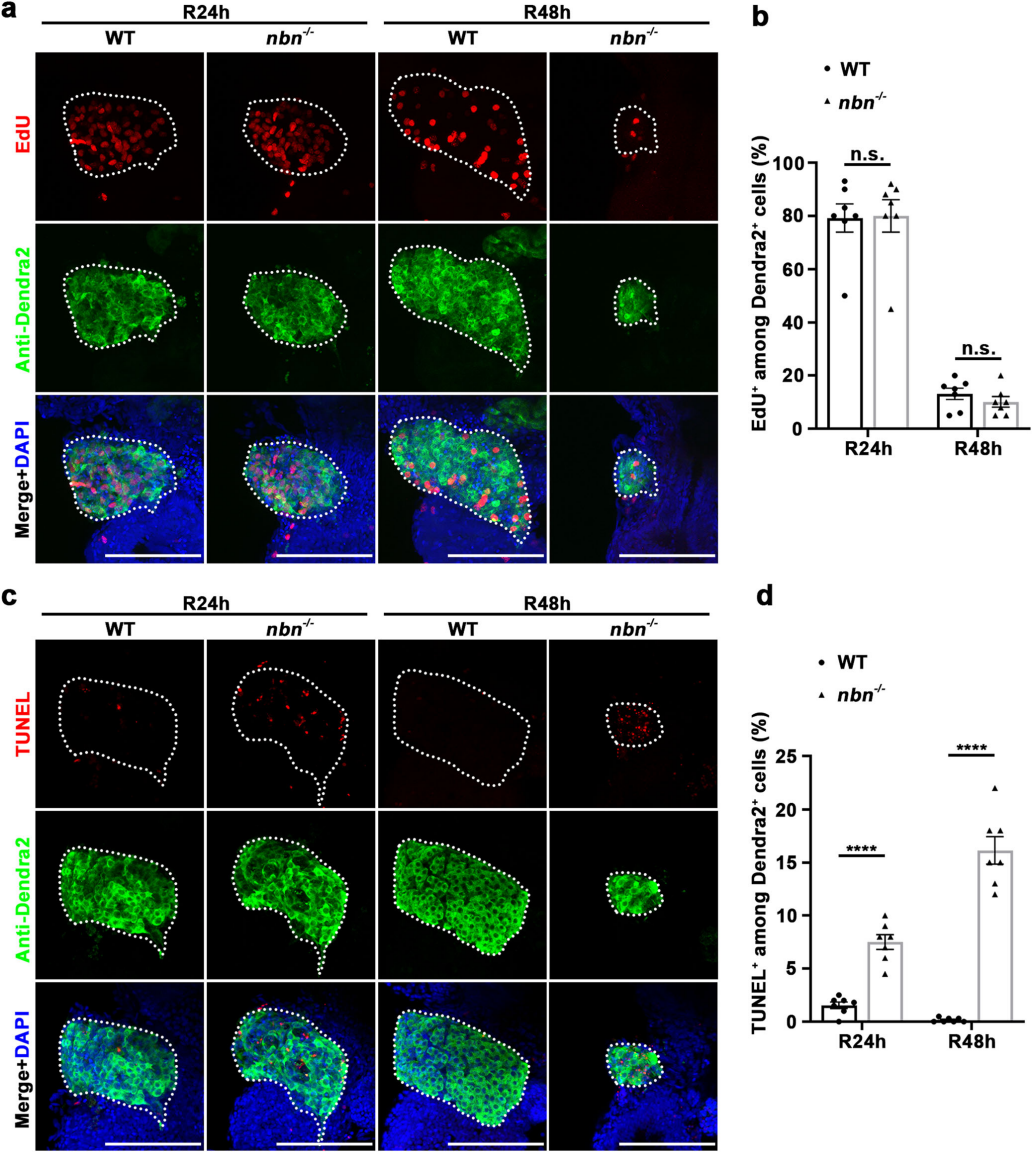
The *nbn* mutant exhibits normal cell proliferation but aberrant cell apoptosis. **a** Confocal z-stack projection images showing the label of EdU (red), Dendra2 (green), and DAPI (blue) in regenerating livers at R24h and R48h in wild-type and *nbn* mutant. **b** Quantification of the percentage of EdU^+^ cells among Dendra2^+^ cells, *n* = 7 larvae per group. **c** Confocal z-stack projection images showing the label of TUNEL (red), Dendra2 (green), and DAPI (blue) in regenerating livers at R24h and R48h. **d** Quantification of the percentage of TUNEL^+^ cells among Dendra2^+^ cells, *n* = 7 larvae per group. Asterisks indicate statistical significance: ****P < 0.0001 using *t*-tests analysis when compared to control. Scale bars, 100 μm; error bars, ±SEM. R regeneration time after the withdrawal of Mtz, WT wild-type, DAPI 4′, 6-diamidino-2-phenylindole, n.s. no significance.

Liver regeneration after extreme hepatocyte loss mainly includes dedifferentiation of BECs into BPPCs and proliferation and redifferentiation of BPPCs into nascent hepatocytes and BECs^8,9^. To investigate how *nbn* affected the maintenance of regenerated hepatocytes, we first detected the expressions of the hepatoblast and BPPC markers forkhead box A3 (*foxa3*), hematopoietically expressed homeobox (*hhex*), and SRY-box transcription factor 9b (*sox9b*), all of which were induced in BPPCs during liver regeneration^14,16^. The transcriptional activation of *foxa3*, *hhex*, and *sox9b* in *nbn* mutant was comparable to wild-type at R0h (Supplementary Fig. 7a), indicating that the process of dedifferentiation is undisturbed. Given that the regenerating liver in *nbn* mutant was relatively normal at R24h but significantly diminished at R48h (Fig. 1b, c), we doubted that the reduced liver regeneration is possibly attributable to decreased cell proliferation or increased cell death. We examined the expression of proliferation markers during liver regeneration. We performed a 5-ethynyl-20-deoxyuridine (EdU) incorporation assay. EdU and Dendra2 double immunostaining results indicated no significant difference in the percentage of EdU and Dendra2 double-positive cells between wild-type and *nbn* mutant larvae at R24h and R48h (Fig. 4a, b). Proliferating cell nuclear antigen (PCNA) immunostaining also displayed that the proportion of PCNA among Dendra2 double-positive cells showed no difference between wild-type and *nbn* mutant larvae (Supplementary Fig. 7b, c). We then performed a TdT-mediated dUTP nick-end labelling (TUNEL) assay to detect cell apoptosis. TUNEL assay showed a significant increase of apoptotic Dendra2 positive cells in *nbn* mutant, compared with wild-type at R24h and R48h (Fig. 4c, d). Above all, these results suggest that loss of *nbn* stimulates cell apoptosis, which accounts for the abrogation of regenerated hepatocytes.

### Accumulative DNA damage and impaired ATR-Chk1 signaling in nascent hepatocytes in *nbn* mutant during liver regeneration

**Fig. 5:**
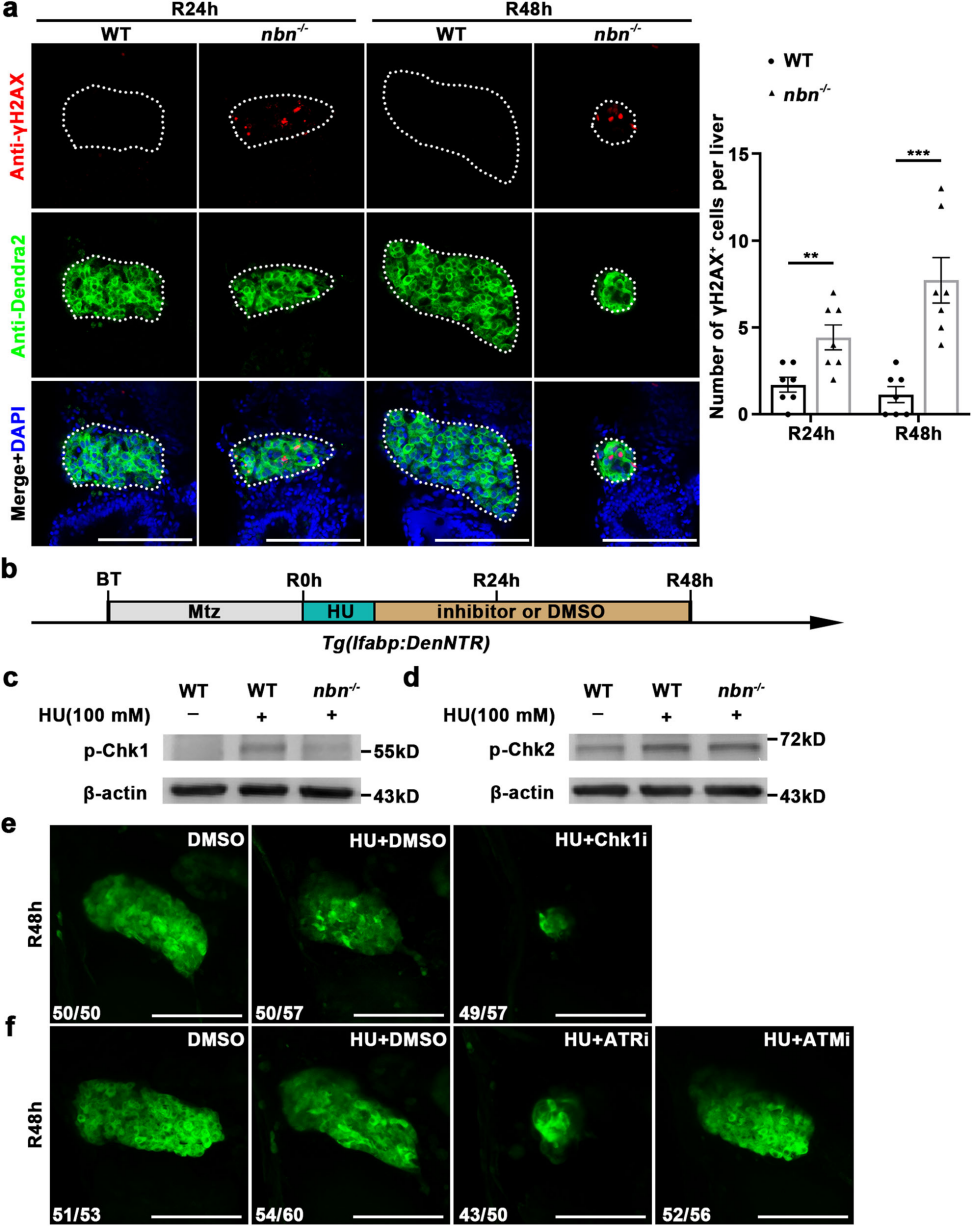
*Nbn* mutation elevates DNA damage and deactivates ATR-Chk1 signaling in liver regeneration. **a** Single optical section images showing γH2AX (red), Dendra2 (green), and DAPI (blue) expressions in regenerating livers at R24h and R48h in wild-type and *nbn* mutant. Quantification of the number of γH2AX^+^ cells per liver, *n* = 7 larvae per group. **b** Schematic illustration of Mtz treatment and inhibitor treatment under HU exposure. **c** Western blot images of p-Chk1 in wild-type, HU-treated wild-type and HU-treated *nbn* mutant larvae. Note that *nbn* mutation abrogates the Chk1 phosphorylation upon HU treatment. **d** Western blot images of p-Chk2 in wild-type, HU-treated wild-type, and HU-treated *nbn* mutant larvae. **e** Confocal projection images showing the regenerating livers of Chk1 inhibitor treatment under the *Tg(lfabp:DenNTR)* background. **f** Confocal projection images showing the regenerating livers of ATR inhibitor and ATM inhibitor treatment under the *Tg(lfabp:DenNTR)* background. Note that ATR inhibitor but not ATM inhibitor treatment shows reduced liver regeneration in wild-type upon HU treatment. Asterisks indicate statistical significance: **P < 0.01; ***P < 0.001 using *t*-tests analysis when compared to control. Numbers indicate the proportion of larvae exhibiting the expression shown. Scale bars, 100 μm; error bars, ±SEM. WT wild-type, R regeneration time after the withdrawal of Mtz, DAPI 4′, 6-diamidino-2-phenylindole, HU hydroxyurea, Chk1i Chk1 inhibitor CHIR-124, ATRi ATR inhibitor VE-821, ATMi ATM inhibitor KU55933.

Nbn is one of the main components of MRN complex, which acts as a central sensor of DSBs and plays a role in preventing replication stress for faithful DNA replication^20,21^. Complete deletion of any component of the MRN complex is incompatible with life in mice, presumably due to replication-associated DSBs^33–36^. To evaluate the level of endogenous DNA damage in *nbn* mutant, we measured the typical DNA damage marker, phosphorylated H2AX (γH2AX). Although γH2AX was rarely detected in siblings, γH2AX positive cells were significantly increased and accumulated in the *nbn* mutant liver at R24h and R48h (Fig. 5a). TUNEL assay combined with γH2AX staining further revealed that cells with accumulated DNA damage undergo apoptosis (Supplementary Fig. 8). The increased number of γH2AX positive cells in *nbn* mutant larvae suggested that disruption of *nbn* leads to accumulation of endogenous DNA damage in regenerating liver during rapid proliferation of hepatocytes.

All living cells are constantly challenged by damaging agents of endogenous and external stresses. Numerous highly conserved DDR pathways exist to activate DNA damage repair and cell cycle checkpoints^37,38^. ATM and ATR are central to the DDR; when activated, both trigger a series of downstream signaling events, including Chk2 and Chk1, respectively^39,40^. In the case of DNA damage in rapidly proliferating cells, we evaluated the activation of DNA damage checkpoints in *nbn* mutant by western blotting. We checked phosphorylation levels of Chk1 and Chk2 in response to HU exposure (Fig. 5b), which could trigger replication fork stalling and induce DNA damage^31,32^. Notably, the phosphorylation level of Chk1 at Ser345 (p-Chk1) in wild-type was increased at R48h after 100 mM HU treatment. However, the activation of p-Chk1 in *nbn* mutant was largely compromised (Fig. 5c and Supplementary Fig. 9a), indicating that disruption of Nbn inhibits the Chk1 signaling during liver regeneration. In contrast, the phosphorylation level of Chk2 at Thr68 (p-Chk2) was comparable in *nbn* mutant and wild-type under HU treatment (Fig. 5d and Supplementary Fig. 9b), indicating that Chk2 signaling remains functional in *nbn* mutant. To verify whether Chk1 plays a role in liver regeneration, we treated the regenerating liver with the Chk1 inhibitor CHIR-124 after HU exposure. The liver size of CHIR-124 treatment was significantly decreased after HU exposure, representing the phenotype of *nbn* mutant (Fig. 5e). Collectively, these data indicate that Nbn deficiency impairs Chk1 signaling in response to the endogenous DNA damage of the regenerating liver.

To better understand the role of DDR in liver regeneration, we examined the role of ATR and ATM signaling with two specific kinase inhibitors, VE-821 and KU55933. *Tg(lfabp:DenNTR)* larvae treated with 5 µM ATR inhibitor (VE-821) from R8h to R48h showed a much smaller liver compared with the control group under HU exposure, while the liver was relatively normal after inhibition of ATM (Fig. 5f). In addition, the treatment with these inhibitors did not lead to apoptosis of hepatocytes during development (Supplementary Fig. 10). Since ATR inhibition and Chk1 inhibition mimicked the phenotype of *nbn* mutant, we inferred that a failure of ATR-Chk1 signal activation mainly caused the liver regeneration defects. Above all, these data suggest that the blockage of the Nbn-ATR-Chk1 axis is associated with the defective regenerated hepatocytes in *nbn* mutant.

### P53 mediates hepatocyte apoptosis in *nbn* mutant during liver regeneration

**Fig. 6:**
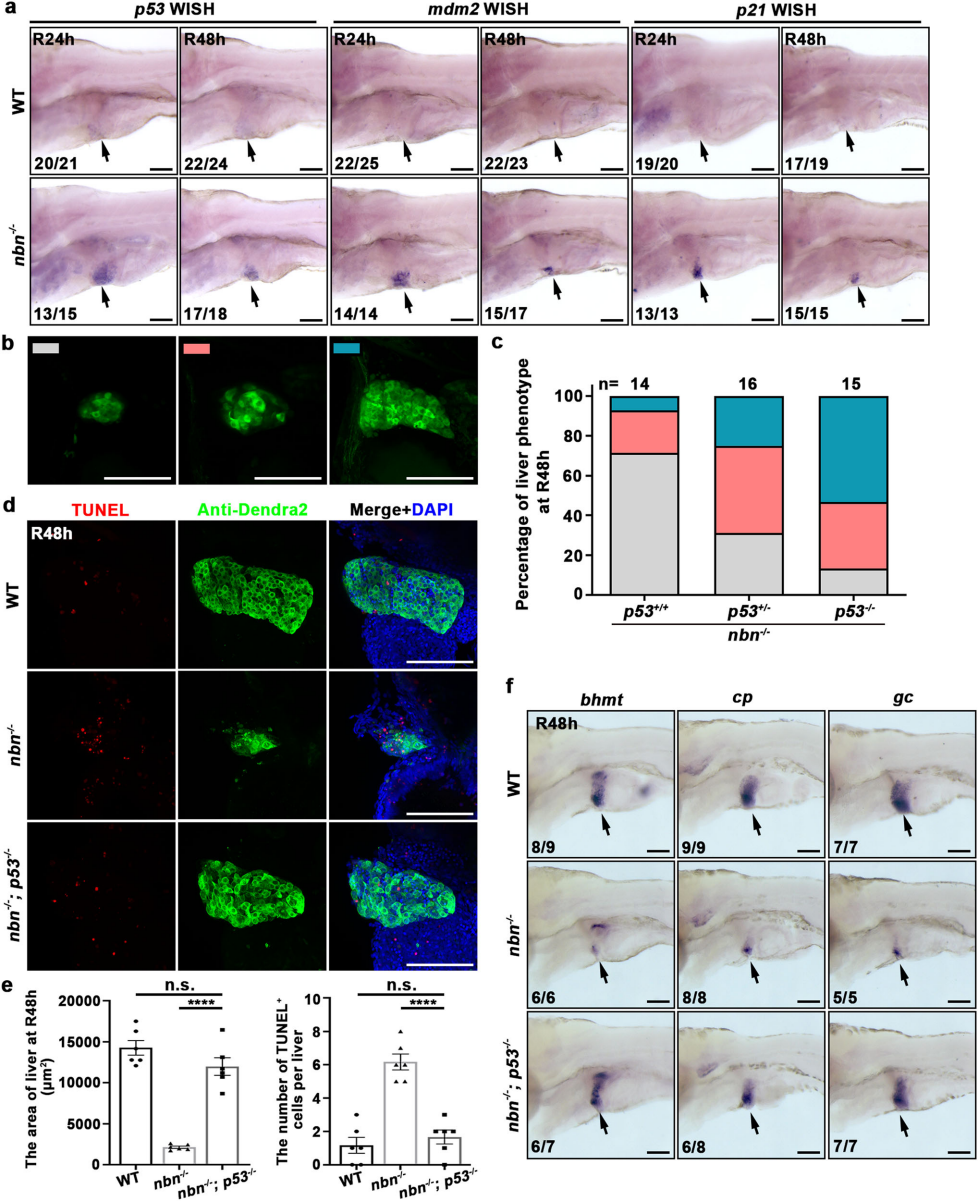
The cell apoptosis of regenerating hepatocytes in the *nbn* mutant partially depends on p53. **a** WISH result showing the expressions of *p53*, *mdm2*, and *p21* in wild-type and *nbn* mutant livers at R24h and R48h (arrows). **b**, **c** Three kinds of standards of liver regeneration **b** and quantitative analysis of regeneration phenotype **c**. Note that inactivation of *p53* can partially rescue the liver regeneration in *nbn* mutant. The numbers of embryos are shown above the columns. **d** Confocal z-stack projection images showing the label of TUNEL (red), Dendra2 (green), and DAPI (blue) in wild-type, *nbn*^*-/-*^, and *nbn*^*-/-*^ *p53*^*-/-*^ double mutant regenerating livers at R48h. Note that cell apoptosis is decreased in *nbn* mutant by introducing *p53*^*-/-*^. **e** Quantification of the area of liver sizes and the number of TUNEL^+^ cells per liver, *n* = 6 larvae per group. **f** WISH images showing the expressions of *bhmt*, *cp*, and *gc* in wild-type, *nbn*^*-/-*^, and *nbn*^*-/-*^ *p53*^*-/-*^ double mutant regenerating livers at R48h (arrows). Asterisks indicate statistical significance: ****P < 0.0001 using *t*-tests analysis when compared to control. Numbers indicate the proportion of larvae exhibiting the expression shown. Scale bars, 100 μm; error bars, ±SEM. R regeneration time after the withdrawal of Mtz, WT wild-type, WISH whole-mount in situ hybridization, DAPI 4′, 6-diamidino-2-phenylindole, n.s. no significance.

As p53 regulates cell cycle progression and apoptosis in response to DNA damage^41–43^, we examined the expressions of several *p53* target genes to check whether the increased cell apoptosis in *nbn* mutant was triggered by aberrant activation of p53. The WISH results showed that the transcription activation of *p53*, *p21*, and *mdm2* were significantly upregulated in *nbn* mutant at R24h and R48h (Fig. 6a). The upregulation of *p21* should result in decreased cell proliferation as previously reported^11^, and then we found the percentage of EdU and Dendra2 double-positive cells was reduced in *nbn* mutant larvae compared with wild-type at R72h and R96h (Supplementary Fig. 11). It implied the reduced proliferation was cumulative in time and appeared in *nbn* mutant during the later stage of liver regeneration. Additionally, the origin of nascent hepatocytes did not change over time and remained from the transdifferentiation of BECs (Supplementary Fig. 12). To further confirm that the upregulation of p53 signaling was responsible for apoptosis in *nbn* mutant, we introduced *tp53*^M214K^ mutant (*p53*^*-/-*^) into the *nbn* mutant, which abrogated normal p53 function in apoptosis^44^. Loss of *p53* did not affect liver regeneration as previously described (Supplementary Fig. 13)^15^. The size of regenerating liver in *nbn* mutant was partially rescued in *p53* heterozygous, and the rescue effect was more obvious in *p53* homozygous mutant (Fig. 6b, c). Further study showed that cell apoptosis was significantly decreased at R48h in *nbn*^*-*/-^ *p53*^*-/-*^ double mutant compared with *nbn* mutant embryos (Fig. 6d, e). Besides, the expressions of hepatocyte markers were well restored in *nbn*^*-*/-^ *p53*^*-/-*^ double mutant compared with *nbn* mutant by the WISH results of *bhmt*, *cp* and *gc* (Fig. 6f). The expression of *p21* was downregulated in *nbn*^*-*/-^ *p53*^*-/-*^ double mutant compared to *nbn* mutant, suggesting that *p21* acts in a p53-dependent manner (Supplementary Fig. 14). In summary, the cell apoptosis of regenerating hepatocytes in *nbn* mutant is p53-dependent.

### The MRN complex is required for the survival of regenerating hepatocytes in zebrafish fibrotic liver model and partial hepatectomy in mice

To study the contribution of MRN complex in other forms of liver regeneration, we applied extreme liver injury in ethanol (EtOH)-induced fibrotic liver zebrafish model^17^. Larvae were exposed to 1.5% EtOH for inducing liver fibrosis (Supplementary Fig. 15a). The *nbn* mutant exhibited liver regeneration defects and increased cell apoptosis in fibrotic liver injury model (Supplementary Fig. 15b and c). These results suggest the MRN complex plays a role in the liver regeneration of the fibrotic liver injury model.

To further extend the significance of our findings, we further checked the roles of MRN complex in liver regeneration using a partial hepatectomy (PHx) model in mice (Supplementary Fig. 16a). We found a reduction in the liver-to-body weight ratio (Supplementary Fig. 16b) and a significant increase of apoptotic hepatocytes (Supplementary Fig. 16c) with the Chk1 inhibitor CHIR-124 after HU exposure. Besides, CHIR-124 treatment increased the activity of serum alanine aminotransferase (ALT) and aspartate aminotransferase (AST) while decreasing serum albumin concentration compared to the control group (Supplementary Fig. 16d). Taken together, these results indicate the MRN complex has a conserved function that regulates the maintenance of nascent hepatocytes in mouse PHx model as in zebrafish.

## Discussion

**Fig. 7:**
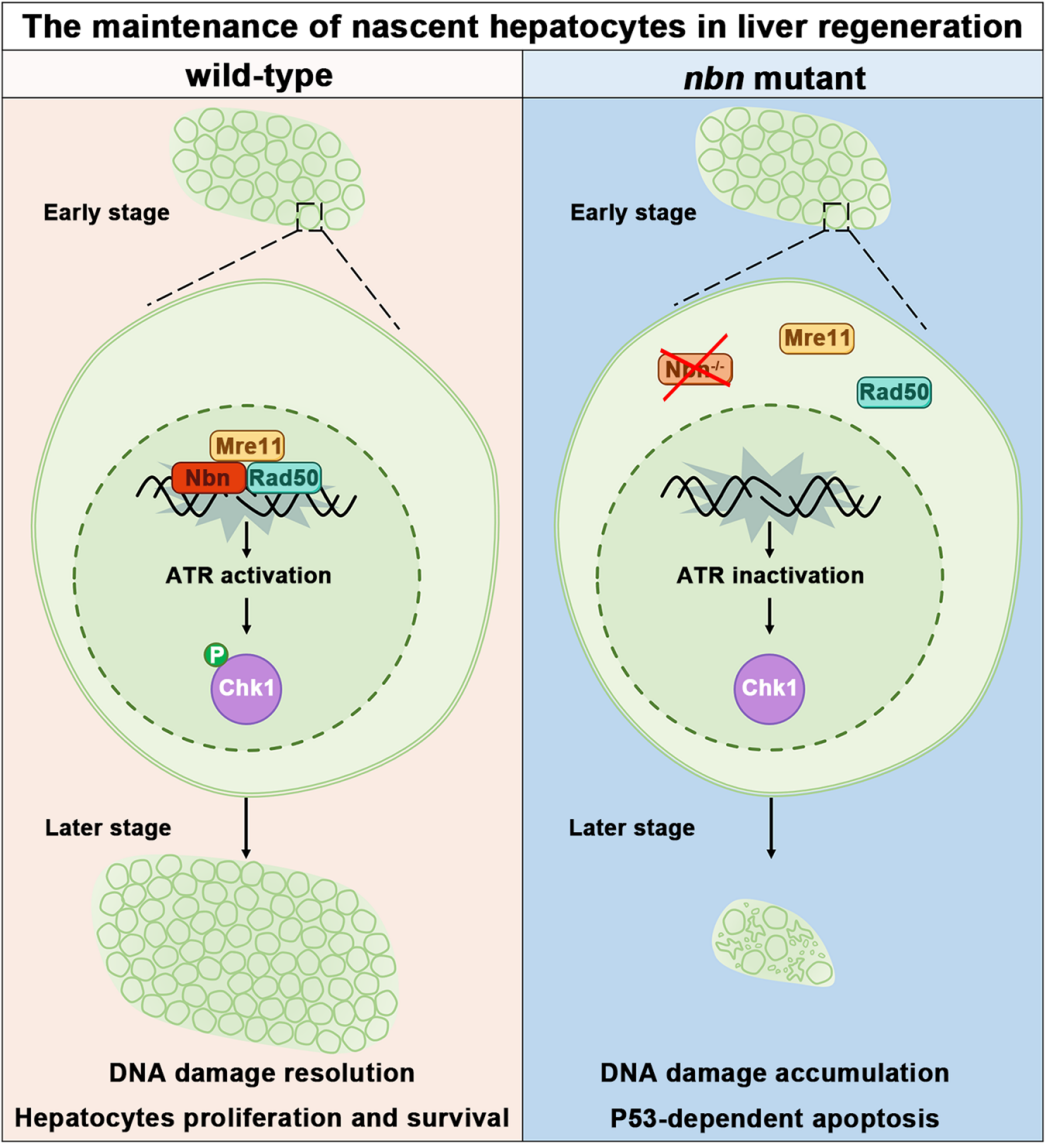
The molecular mechanism model showing the MRN complex governs the survival of regenerating hepatocytes in the later stage of liver regeneration. During the highly proliferative phase of liver regeneration, the hepatocytes undergo endogenous DNA damage caused by replication stress. In normal livers, the MRN complex is recruited to DNA damage and activates ATM and ATR, subsequently triggering an intricate signal transduction network called DNA damage response. Activated cell cycle checkpoints arrest cell cycle progression and allow DNA repair so regenerated hepatocytes can survive and proliferate. In *nbn* mutant, the truncated Nbn mislocalizes in the cytoplasm, and the MRN complex cannot be recruited to the DNA damage site. The ATR-Chk1 signaling is prevented from activation. The stalled replication fork may collapse and trigger p53-dependent apoptosis, ultimately leading to defective liver regeneration.

During liver regeneration, BPPCs undergo rapid cell proliferation and inevitably face an increased risk of replication-associated spontaneous DNA damage and stalled replication fork, referred to as DNA replication stress, which is required to be efficiently resolved by robust DNA damage response (DDR). Cell cycle arrest is required to provide time for DNA repair and replication fork restart; If fork restart fails, replication forks collapse into DSBs^45–48^. The function of DDR in liver regeneration after severe liver injury remains largely unknown. Here, using a zebrafish extreme liver injury model with an ENU-based forward genetic screen, we identified a novel mutant with relatively normal liver development but exhibiting severe liver regeneration defects, thus revealing previously unreported roles of DDR factor *nbn*. Mechanically, recruitment of Nbn can activate ATR kinase activity and then phosphorylate downstream target Chk1. Activated Chk1 promotes replication forks stabilization and restart during liver regeneration so that the regenerated hepatocytes can survive. Moreover, we described that disrupting the function of Rad50 and Mre11a mimics the phenotype of *nbn* mutant. Taken together, we uncovered that *nbn* activates the ATR-Chk1 axis through MRN complex, which is indispensable for the survival of regenerated hepatocytes (Fig. 7).

Traditionally, the MRE11/RAD50/NBN deficiency causes chromosome instability syndromes in humans^28,49,50^. The patient may display growth retardation, immunodeficiency, and predisposition to cancer. Cells from patients exhibited increased sensitivity to ionizing radiation and increased levels of spontaneous chromosomal fragility^33,49,51^. In mice, the disruption of either component of the MRN complex causes early embryonic lethality, which makes it difficult to uncover the disease-associated pathology and related molecular mechanism in the mouse model^34,36,52^. With in-depth analysis during zebrafish development, we found the larvae of *nbn* or *rad50* mutants exhibited relatively normal morphological appearance. To test whether the homozygous mutant animals can survive to adulthood, we genotyped the progenies generated from heterozygous parents at 1 month post-fertilization (mpf) and 4 mpf. Homozygous mutants could be genotyped at 1 mpf but completely failed to survive to 4 mpf. Intriguingly, we also found that the *nbn* and *rad50* mutant zebrafish showed significant growth retardation relative to siblings, as zebrafish *mre11a* mutant showed growth defects and failed to survive to adult stages^53^. That is consistent with human Nijmegen Breakage Syndrome. Thus, our findings suggest that Nbn or Rad50 deficiency zebrafish may afford an opportunity to understand the mechanism of chromosome instability syndromes in humans.

MRN complex is recognized as one of the first sensors that respond to DSB or replication stress. Stimulated by DSB or replication fork arrest, MRN complex recruit and activate ATM and ATR kinases. Then, Chk2 and Chk1 are phosphorylated to coordinate DNA repair and cell cycle progression^22^. Studies on *nbn* mutants showed that DNA damage accumulation and apoptosis increase in the regenerated hepatocytes, which is related to the failed MRN/ATR/Chk1 axis activation. During the rapid proliferative phase of liver regeneration, the importance of DDR for nascent hepatocyte survival is further emphasized by hydroxyurea treatment.

In our study, treatment with selective inhibitors of MRN/ATR/Chk1 axis did not induce hepatocyte apoptosis during zebrafish development, indicating that these small molecules were non-toxic at the doses we used. These small molecules have been verified to be highly selective previously^54^ and were used at concentrations of 10 μM of ATMi and ATRi in cancer cells^55^. However, studies of ATM/ATR inhibitors in zebrafish remain limited. Therefore, we explored the dosages of ATMi and ATRi in zebrafish and finally determined the appropriate doses for treating the larvae with 2 μM and 5 μM, respectively. However, due to the general disadvantage of small molecules of lacking exclusive selectivity, additional genetic validation or further mechanistic studies are needed.

Furthermore, using a zebrafish fibrotic liver model, we found the *nbn* mutants exhibited liver regeneration defects and increased hepatocyte apoptosis. Similarly, using a mouse partial hepatectomy model, we also found a defective hepatic regeneration and a significant increase of apoptotic hepatocytes with inhibition of the function of MRN complex. These results indicate that the role of MRN complex is also involved in maintaining the survival of nascent hepatocytes in other forms of liver injuries, indicating the critical role of MRN complex in a broad manner of liver regeneration.

Upon activation of the DDR, p53 is activated to induce cell cycle arrest, DNA repair, or apoptosis^41–43^. How p53 chooses the outcomes between the competing cell survival and cell death upon the extent and type of DNA damage and cell type has been a great fascination but is not yet entirely elucidated. Here, we describe that the p53-p21 axis was hyperactivated in *nbn* mutants. Loss of p53 could partially rescue the deficiency of liver regeneration in *nbn* homozygous mutants. These results suggest that p53 loss of function benefits the survival of regenerated hepatocytes in *nbn* deficiency zebrafish. Understanding detailed molecular mechanisms underlying the regulation of MRN-mediated p53 activation awaits further investigation.

Collectively, this study revealed a novel and vital role of MRN-ATR-Chk1 signaling in regenerated hepatocytes after extreme liver injury. Loss of *nbn* did not affect the early phase of BEC-mediated transdifferentiation but specially regulated the survival of nascent hepatocytes through the ATR-Chk1 axis. *rad50* mutants validated the phenotype of *nbn* mutant is a direct consequence of MRN complex dysfunction. Given the conservation roles of BECs upon severe liver injury from zebrafish to mammals, the MRN complex may be a potential target for therapeutics after severe liver injury.

## Methods

### The agency or committee that granted approval

All animals are housed in temperature- and light-controlled facilities and are maintained in accordance with the Guide for Care and Use of Laboratory Animal and the Animal Welfare Act. Experiments were performed with the approval of the Institutional Animal Care and Use Committee (IACUC) at Southwest University.

### Zebrafish strains

Zebrafish strains were raised and maintained under standard laboratory conditions according to Institutional Animal Care and Use Committee protocols. All experimental protocols were approved by Southwest University (Chongqing, China). The transgenic or mutant zebrafish lines *Tg(lfabp:Dendra2-NTR)*^*cq1*^ abbreviated as *Tg(lfabp:DenNTR)*^*cq1*^, *Tg(Tp1:Tomato)*, *Tg(krt18:CreER)*^*cq74*^*, Tg(βactin:loxP-DsRed-loxP-GFP)*^*s928*^, *Tg(lfabp:loxP-STOP-loxP-DsRed)*^*cq4*^*, Tg(hsp70l:nbn-p2A-mCherry)*^*cq141*^, *Tg(hsp70l:Myc-nbn*^*WT*^*)*^*cq142*^, *Tg(hsp70l:Myc-nbn*^*cq137*^*)*^*cq143*^,*nbn*^*cq137*^ (*lvv*) mutant, *nbn*^*cq139*^ mutant, *rad50*^*cq138*^ mutant, *rad50*^*cq140*^ mutant, *tp53*^M214K^ mutant, and the polymorphic line SJD were used in this study. A complete list of zebrafish strains is provided in Table S1. All the strains are used as stable, germline transgenic lines in this study. Embryos were treated with 0.003% 1-phenyl-2-thiourea (PTU, Sigma-Aldrich) to inhibit pigment formation. Larvae were anesthetized in 0.16 g/L tricaine (Sigma-Aldrich) prior to experiments.

### ENU mutagenesis and positional cloning of the *lvv* mutant

ENU mutagenesis was carried out as previously described^56^ for mutations affecting liver regeneration. Briefly, the adult male zebrafish of *Tg(lfabp:DenNTR)*^*cq1*^ were treated with 3.5 mM ENU for 1 h at weekly intervals and repeated for six times. Two weeks after the ENU treatment, those male zebrafish were outcrossed to AB females to generate F1 families. Then F1 fish were mated to *Tg(lfabp:DenNTR)*^*cq1*^ to generate F2 families. F2 sibling were intercrossed to generate F3 embryos for screening. The morphology of regenerating livers was analyzed at R48h after liver ablation with Mtz treatment in F3 embryos.

Heterozygous *lvv* fish were crossed with the polymorphic line SJD to generate the mapping population. Positional cloning was carried out as previously described^56–58^. The mutation was mapped to chromosome 16 by bulk segregation analysis with simple sequence length polymorphism (SSLP) markers Z6021 and Z3633. Through analysis of 935 mutants, the mutation was finally flanked by two designed SSLP markers, L16m1 (7 recombinants) and L16m2 (1 recombinant). The location of the *lvv* mutation was narrowed to a 0.5 million base pairs (Mb) genomic region. The coding sequences of candidate genes were sequenced by sibling and *lvv* mutant embryos, and the putative mutation was confirmed by genomic DNA sequencing. A complete list of the primers is provided in Table S2.

### Generation of the *nbn*^*cq139*^ and *rad50*^*cq140*^ mutant lines

The *nbn*^*cq139*^ and *rad50*^*cq140*^ mutant lines were generated by CRISPR/Cas9 technology, as previously described^59^. In brief, gRNA (50 pg) and Cas9 mRNA (300 pg) were co-injected into 1-cell stage wild-type embryos, and the lysate of about 10 embryos at 48 hpf was used as templates for PCR. The PCR products were sequenced to examine potential indels that occurred in *nbn* and *rad50* gRNA target region, and embryos with effective genome editing were raised into adults (F0). Then, F0 fish were screened to identify the founder whose progeny carried the indels, and offspring of identified F0 was raised up to get the candidate progeny (F1). Last, individual F1 adults were screened by PCR using the tail fin genomic DNA, and the genotypes of mutants were determined by DNA sequencing. The target sequence of *nbn* and *rad50* and PCR primers are depicted in Table S2.

### Generation of transgenic lines

Zebrafish *nbn* full-length coding sequence was cloned from 48 hpf cDNA. The primers for full-length cloning and site-directed mutagenesis are depicted in Table S2. *Tg(hsp70l:nbn-p2A-mCherry; cryaa:Venus)*^*cq141*^, *Tg(hsp70l:Myc-nbn*^*WT*^*; cryaa:Venus)*^*cq142*^, and *Tg(hsp70l:Myc-nbn*^*cq137*^*; cryaa:Venus)*^*cq143*^ lines were produced using the pBluescript vector. Constructs flanked by the I-SceI (NEB) restriction sites were co-injected with I-SceI into zebrafish embryos of the *Tg(lfabp:DenNTR)*^*cq1*^ background at the 1-cell stage for transgenesis. Larvae were screened for expression of the yellow fluorescent protein (Venus) in the eyes. *Tg(hsp70l:nbn-p2A-mCherry; cryaa:Venus)*^*cq141*^ expressed mCherry in the whole body upon heat shock. For *Tg(hsp70l:Myc-nbn*^*WT*^*; cryaa:Venus)*^*cq142*^ and *Tg(hsp70l:Myc-nbn*^*cq137*^*; cryaa:Venus)*^*cq143*^, Myc-tagged Nbn^WT^ or Nbn^137^ was verified by antibody staining. These zebrafish were raised and crossed to identify founder fish with germline integration, from which stable transgenic lines were established.

### *Whole-m*ount in situ hybridization and antibody staining

Whole-mount in situ hybridization were performed as previously described^15,60^ using the *bhmt*, *cp*, *gc*, *prox1a*, *fabp2*, *trypsin*, *insulin*, *foxa3*, *hhex*, *sox9b*, *nbn*, *rad50*, *p53*, *mdm2*, and *p21* probes. A complete list of the primers is provided in Table S2. cDNA from the whole embryos at 3 dpf, R0h and R48h were used as a template for PCR to amplify the genes. Digoxygenin (Dig) labeled antisense probes were synthesized from PCR products using the T7 or SP6 RNA polymerase (Roche). Larvae were fixed with 4% paraformaldehyde (PFA) in PBS at 4 °C overnight, followed by dehydration and storage in 100% methanol at -20 °C for at least 24 h. Then the larvae were continuously rehydrated from 75%, 50%, 25%, to 0% methanol in PBT (0.1% Tween in PBS). After five times wash with PBT in 1 h, the larvae were prehybridized in HYB (50% formamide, 5 × SSC, 0.1% Tween-20, 5 mg/ml torula yeast RNA, 50 mg/ml heparin) at 67 °C for 3–5 h. Replace the HYB with Dig-labeled probes diluted in HYB and hybridize overnight at 67 °C. Remove the probes and wash successively with 100%, 75%, 50%, 25%, and 0% HYB in 2 × SSCT at 67 °C, and finally with 0.2 × SSCT. Afterward, the larvae were serially replaced with 25%, 50%, 75%, and 100% MABT (150 mM maleic acid, 100 mM NaCl, 0.1% Tween-20, pH 7.5) in 0.2 × SSCT, and blocked for 2 hours with 2% Block Reagent (Roche) in MABT. The larvae were then incubated with anti-Dig-AP antibody (Roche) at a dilution of 1:1000 with blocking buffer at 4 °C overnight. Remove the antibody and rinse the larvae with MABT for eight times. At last, the larvae were stained with NBT/BCIP solution (Roche). Images were captured using a SteREO DiscoveryV20 microscope equipped with AxioVision Rel 4.8.2 software (Carl Zeiss).

Zebrafish whole-mount antibody staining was carried out as previously described^61–64^. The skin of larvae was manually removed with tweezers. After five times wash with PT (1% Triton X-100 in PBS), the larvae were incubated at 4 °C overnight with the following primary antibodies: Dendra2 (1:500, ABIN361314, Antibody-online), Dendra2 (1:500, TA180094, Origene), PCNA (1:500, ab29, Abcam), γH2AX (1:500, GTX127342, Gene Tex), 2F11 (1:1000, ab71286, Abcam), GFP (1:1000, ab6658, Abcam), mCherry (1:500, ARG55723, ArigoBio), DsRed2 (1:1000, sc101526, Santa Cruz), and Myc (1:1000, ab9106, Abcam). The primary antibodies were diluted in the blocking solution (PBS + 4% BSA + 1% Triton X-100). Then, the larvae were washed eight times with PT and incubated with Alexa fluorescent-conjugated secondary antibodies diluted in the blocking solution: donkey anti-rabbit IgG Alexa fluor 488-conjugated (1:1000, A21206, Invitrogen), donkey anti-rabbit IgG Alexa fluor 647-conjugated (1:1000, A31573, Invitrogen), donkey anti-mouse IgG Alexa fluor 568-conjugated (1:1000, A10037, Invitrogen), donkey anti-mouse IgG Alexa fluor 647-conjugated (1:1000, A31571, Invitrogen), donkey anti-goat IgG Alexa fluor 568-conjugated (1:1000, A11057, Invitrogen), and donkey anti-goat IgG Alexa fluor 633-conjugated (1:1000, A21082, Invitrogen). After five washes with PT, the larvae were mounted and imaged. Images were captured using ZEN 2010 software equipped on an LSM780 or LSM880 confocal microscope (Carl Zeiss).

### EdU labeling and TUNEL assay

The EdU labeling and TUNEL assay were performed as previously described^8,62,65^. In brief, we injected the EdU mixture (containing 0.2 mM EdU, 2% DMSO, and 4% phenol red) into the hearts of larvae and fixed the sample with 4% PFA after 1-h post-injection followed by using the Click-iT EdU Kit (Invitrogen) to label the proliferation cells. For terminal deoxynucleotidyl transferase-mediated dUTP nick end labeling (TUNEL) assay, embryos at R48h were fixed with 4% PFA. After methanol dehydration, rehydration, Proteinase K digestion, and acetone treatment, the embryos were stained with the In Situ Cell Death Detection Kit, TMR Red (Roche) at 37 °C for 2 h as described by the manufacturer.

### Chemical treatment, heat shock, and BODIPY assay

For Mtz treatment, the *Tg(lfabp:DenNTR)* transgenic larvae at 5 dpf were incubated with 10 mM Mtz (metronidazole, Sigma) for 24 hours. For ethanol (EtOH) treatment, larvae were treated with 1.5% EtOH (Sigma Aldrich) for 48 hours, starting from 4 dpf^17^. For HU (hydroxyurea, Sigma) treatment, embryos were treated with 100 mM HU in egg water from R0h to R8h. Larvae were treated with 0.1 μM Chk1i (CHIR-124, Selleck), 5 μM ATRi (VE-821, Selleck), 2 μM ATMi (KU-55933, Selleck) and 20 μM Mirin (Selleck) from R8h to R48h. The chemical solutions were renewed every 24 h to maintain the pharmacological effects. And a 0.2% DMSO solution in egg water was used as a control. When chemical treatment is removed, the larvae need to be washed and recovered in the egg water.

For temporal control of CreER activities, larvae were collected and incubated with 5 μM 4-hydroxytamoxifen (4-OHT, Sigma) for 24 h from 4 to 5 dpf, followed by three washes with egg water.

The larvae of *Tg(hsp70l:nbn-p2A-mCherry)*^*cq141*^ were heat-shocked at 38.5 °C for 40 min at the indicated time frame and returned to 28.5 °C for further analysis.

For BODIPY feeding assay, larvae were fed with BODIPY FL C5 (Invitrogen) for 4 h at 5 dpf and then live imaging, as previously described^26^.

### Western blotting

The western blotting was carried out as previously described^61^. In brief, total protein was extracted from wild-type and *nbn* mutant embryos (30 larvae per group) using RIPA lysis buffer containing protease inhibitor, phosphatase inhibitor and PMSF. The protein samples were separated on 10% SDS-PAGE gel and then transferred to a 0.45 μm PVDF membrane (Millipore). The membranes were blocked in 5% BSA for 2 h at the room temperature and immunoblotted with the primary antibodies as follows: β-actin (1:2000, RLM3121, Ruiying Biological), Phospho-Chk1 (Ser345) (1:1000, 2348, Cell Signaling), and Phospho-Chk2 (Thr68) (1:1000, 2197, Cell Signaling). After incubation with corresponding horseradish peroxidase-conjugated secondary antibodies, SuperSignal West Pico Chemiluminescent Substrate (34577, Thermo Fisher Scientific) was used to visualize via chemiluminescence. All blots derive from the same experiment and that they were processed in parallel.

### Mouse studies

C57BL/6 J mice were purchased from GemPharmatech Company (Nanjing, China). Animals were housed in standard pathogen-free conditions under 12 h day/night cycle and were allowed ad libitum access to water and a standard diet (XieTong Biotech, Nanjing, China). Eight-week-old female mice were given daily intraperitoneal injections of HU (200 mg/kg of body weight) or a combination of HU and Chk1i (10 mg/kg of body weight) for 8 days. On the fourth day, mice were anesthetized with isoflurane and subjected to 70% partial hepatectomy. The weight of regenerated livers as well as the whole body was measured at 4 days post-PHx. The ratio of wet weight of the regenerated liver remnants after PHx over the weight of the whole animal was taken as the liver-to-body weight ratio. At the end of the animal experiment, mice were anesthetized to collect blood and liver tissue and then euthanized.

For serum analysis, blood was allowed to coagulate for 2 h at 4 °C and serum was collected after centrifugation at 2000 rpm for 10 min at 4 °C and stored at −80 °C. Hepatic function was analyzed by commercial kits of aspartate aminotransferase (ab263882, Abcam, UK), alanine transaminase (RDR-ALT-Mu, Reddot Biotech, Canada), and albumin (E-90AL, Immunology Consultants Laboratory, USA) according to the manufacturer’s instructions. Data were acquired using a Spark microplate reader (Tecan, Switzerland).

For immunofluorescence staining, the liver tissues were fixed in 4% PFA for 1 h and transferred to PBS, then to 30% sucrose for 1 day, embedded in O.C.T. compound (Tissue Tek, Sakura) and sectioned at a thickness of 10 µm using a cryomicrotome (CM 1950, Leica). Antigen retrieval was performed for 10 min in Tris/EDTA buffer (10 mM Tris-HCl, 1 mM EDTA, 0.05% Tween-20, pH 9.0) at 95 °C. Cell apoptosis was measured using the In Situ Cell Death Detection Kit (Roche) following the manufacturer’s instructions. Then sections were stained with primary antibody against HNF4α (1:50, SC6556, Santa Cruz, Dallas, TX) and compatible fluorescently labeled secondary antibodies (Invitrogen, 1:500 dilution).

### Quantification and statistical analysis

Unpaired two-tailed Student’s t-test was used for statistical analysis by GraphPad Prism 8; *P* < 0.05 was considered statistically significant. Quantitative data were shown as means ± SEM.

### Reporting summary

Further information on research design is available in the Nature Research Reporting Summary linked to this article.

## Supplementary information

Supplementary Information

## Data Availability

The data that support the findings of this study are available from the corresponding author upon reasonable request.
