# Supplementary material for: The MRN complex maintains the biliary-derived hepatocytes in liver regeneration through ATR-Chk1 pathway
Source: NPJ Regen Med. 2023 Apr 6;8:20. doi: 10.1038/s41536-023-00294-3 (PMC10079969; doi:10.1038/s41536-023-00294-3)
Supplement: Supplementary file 1 — Supplementary Information [file 41536_2023_294_MOESM1_ESM.pdf]

Supplementary Information

Supplementary Figures and Legends

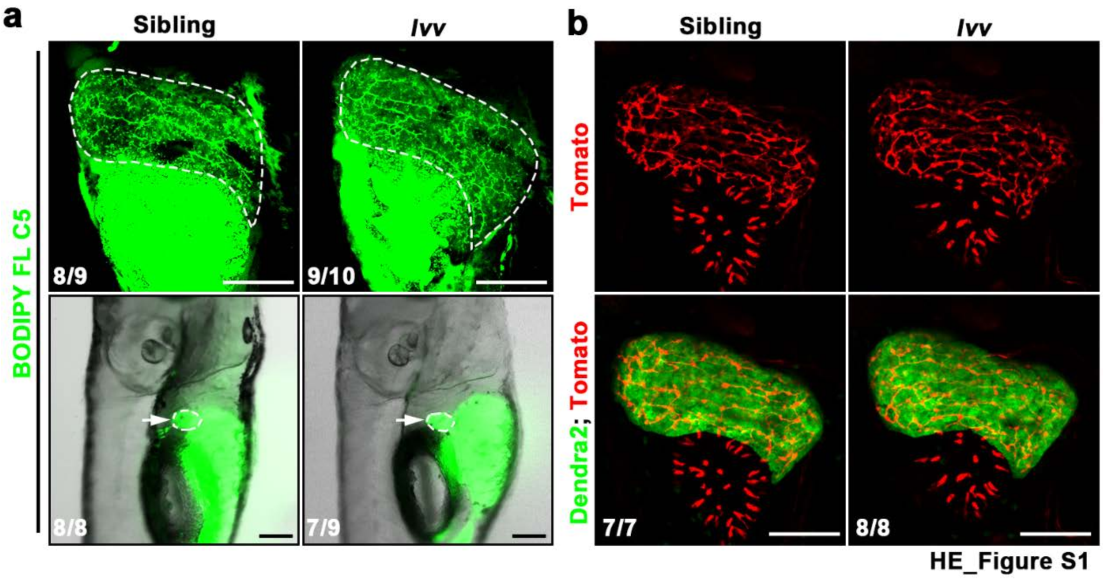

Supplementary Figure 1. The biliary duct system is functional in *lvv* mutant.

a Confocal projection images showing the BODIPY FL C5 (green) in liver and gallbladder (arrows) of sibling and *lvv* mutant at 5 dpf. **b** Confocal projection images showing the Tomato (red) and Dendra2 (green) expressions at 5 dpf using *Tg(Tp1:Tomato; lfabp:DenNTR)* transgenic line. Scale bars, 100 µm. Numbers indicate the proportion of larvae exhibiting the expression shown.

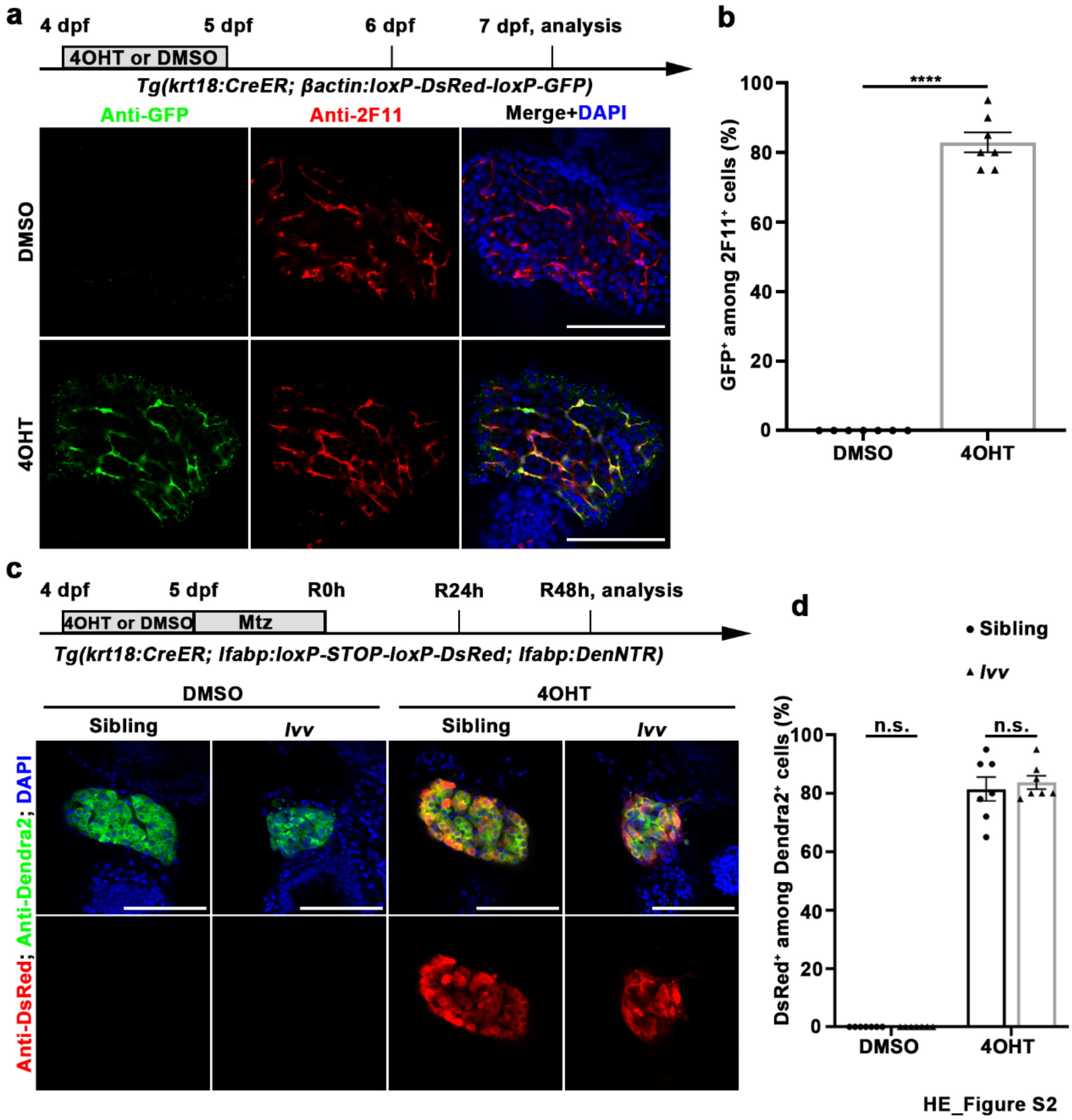

Supplementary Figure 2. Nascent regenerating hepatocytes are derived from BECs after extreme hepatocyte ablation in *lvv* mutant.

a Schematic illustration of the 4OHT or DMSO treatment from 4 dpf to 5 dpf and analysis at 7 dpf in *Tg(krt18:CreER; βactin:loxP-DsRed-loxP-GFP)* transgenic line. Single optical section images showing the staining of GFP and 2F11 in liver at 7 dpf. Note that the expression of GFP merged with 2F11 after 4OHT treatment. **b** Quantification of the percentage of GFP^+ cells among $2F11^+$ cells, n = 7 larvae per group. **c** Schematic illustration of the 4OHT or

DMSO treatment and analysis at R48h after Mtz treatment in *Tg(krt18:CreER; lfabp:loxP-STOP-loxP-DsRed; lfabp:DenNTR)* transgenic line. Single optical section images showing the expressions of DsRed and Dendra2 in regenerating livers at R48h in sibling and *lvv* mutant livers. **d** Quantification of the percentage of $DsRed^{+}$ cells among $Dendra2^{+}$ cells, n = 7 larvae per group. Asterisks indicate statistical significance: ****$P < 0.0001$ using t-tests analysis when compared to control. Scale bars, 100 μm; error bars, ±SEM. Abbreviations: R, regeneration time after the withdrawal of Mtz; DAPI, 4’, 6-diamidino-2-phenylindole; dpf, days post-fertilization; n.s., no significance.

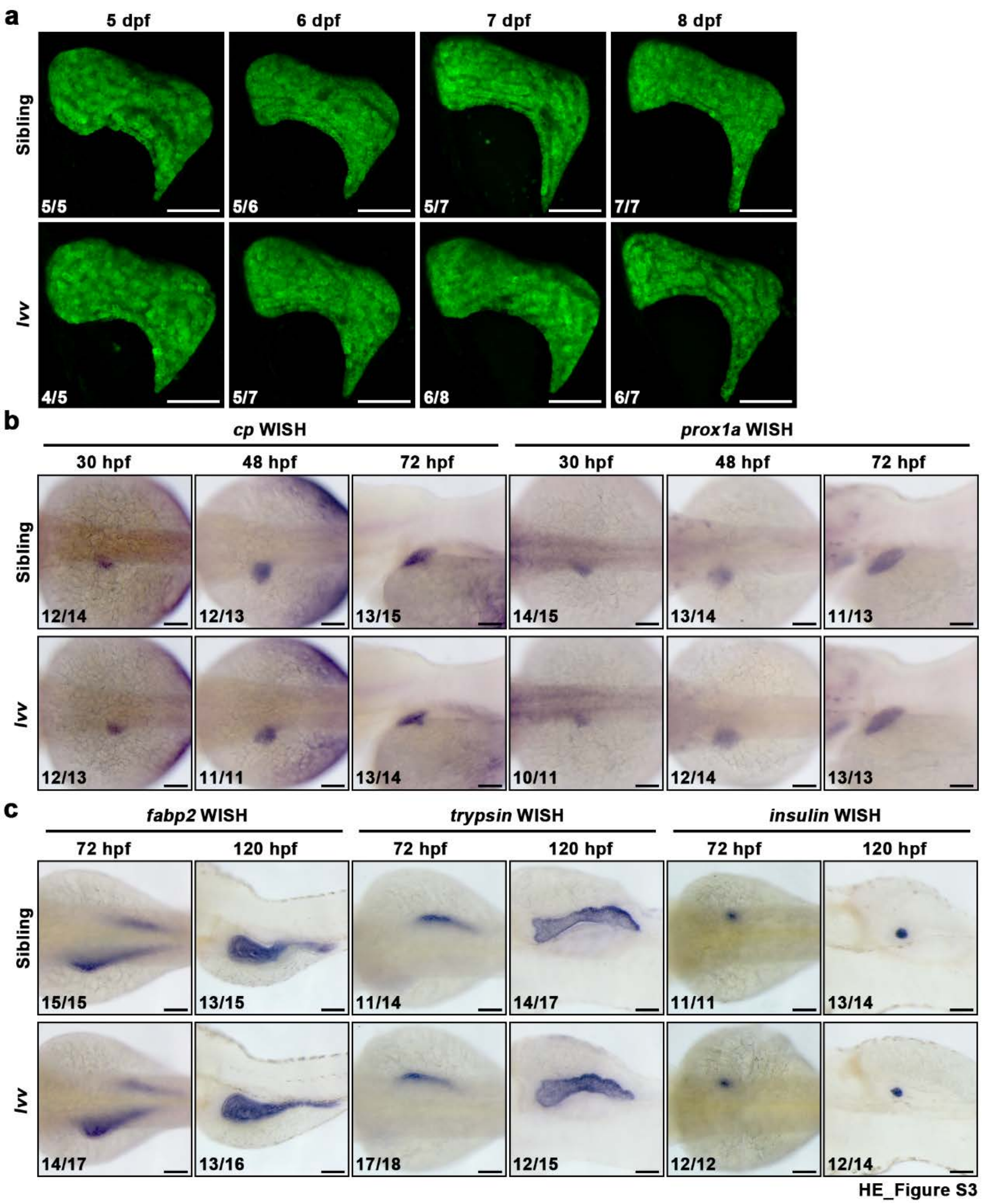

Supplementary Figure 3. The development of digestive organs is normal in *lvv* mutant.

a Confocal projection images showing liver development in sibling and *lvv* mutant from 5 dpf to 8 dpf. **b** WISH images showing the expressions of *cp* and *prox1a* at 30 hpf (dorsal view), 48 hpf (dorsal view), and 72 hpf (lateral view) in sibling and *lvv* larvae. **c** WISH images showing the expressions of *fabp2*,

trypsin, and *insulin* at 72 hpf (dorsal view) and 120 hpf (lateral view) in sibling and *lvv* larvae. Numbers indicate the proportion of larvae exhibiting the expression shown. Scale bars, 100 μm. Abbreviations: WISH, whole-mount *in situ* hybridization, dpf, days post-fertilization.

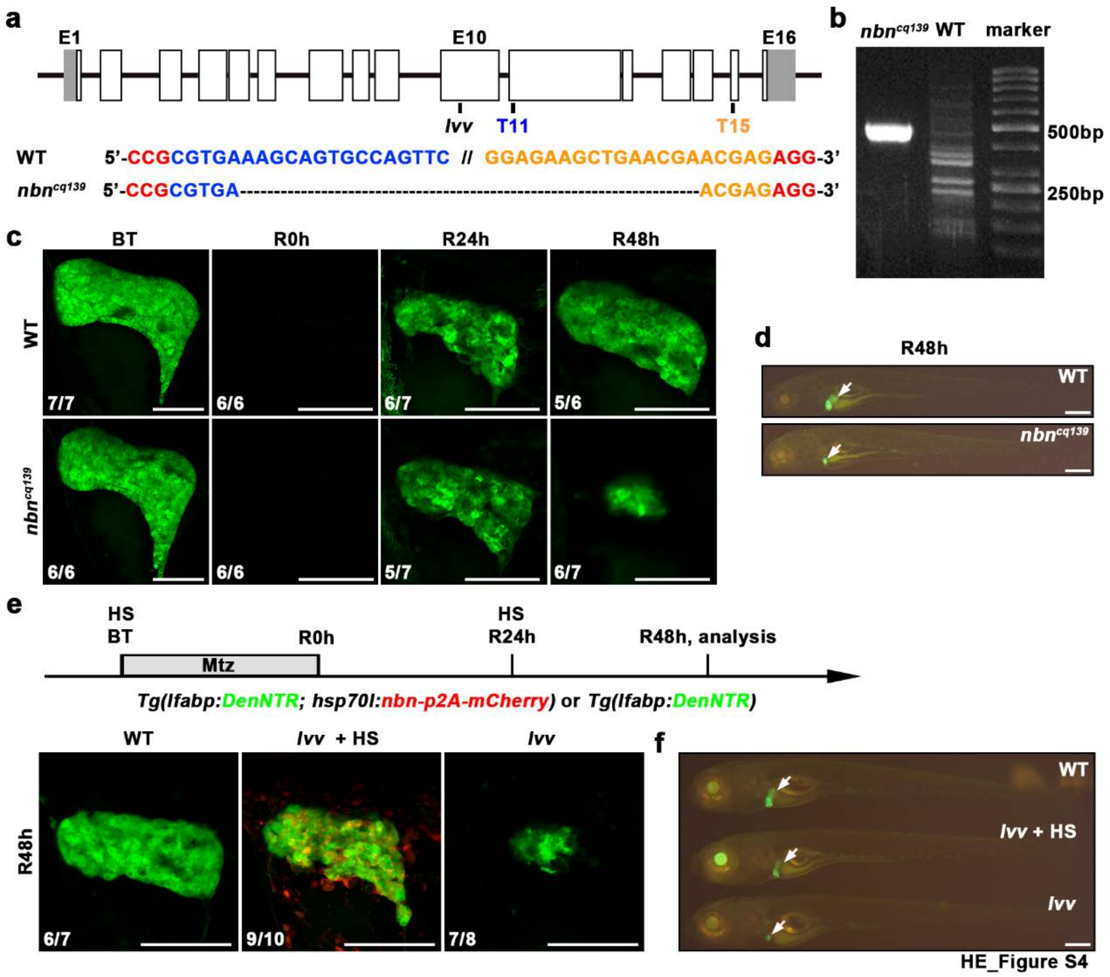

Supplementary Figure 4. Nbn is required for liver regeneration in zebrafish.

a Diagram showing the genomic structure of *nbn* (top panel) and the alignment of gRNA target sequences in *nbn* exon 11 (blue) and exon 15 (orange) (bottom panel). Note that two gRNAs are assembled for large fragment deletion. **b** Agarose gel electrophoresis showing genotyping results of *nbn*^{cq139} and wild-type. **c** Confocal projection images showing the regenerating livers of wild-type and *nbn*^{cq139} mutant from BT to R48h under the *Tg(lfabp:DenNTR)* background. **d** Epifluorescence images showing the regenerating livers (arrows) of wild-type and *nbn*^{cq139} larvae at R48h. **e**

Schematic illustration of Mtz and heat shock treatment and analysis at R48h (top panel). Confocal projection images showing the expressions of Dendra2 (green) and Nbn-p2A-mCherry (red) at R48h (bottom panel). Note that the size of regenerating liver in *lvv* mutant is primarily rescued by Nbn overexpression. **f** Epifluorescence images showing the morphological appearance and regenerating liver (arrows). Numbers indicate the proportion of larvae exhibiting the expression shown. Scale bars, 100 μm (**c** and **e**) and 300 μm (**d** and **f**). Abbreviations: BT, before Mtz treatment; R, regeneration time after the withdrawal of Mtz; WT, wild-type; HS, heat shock.

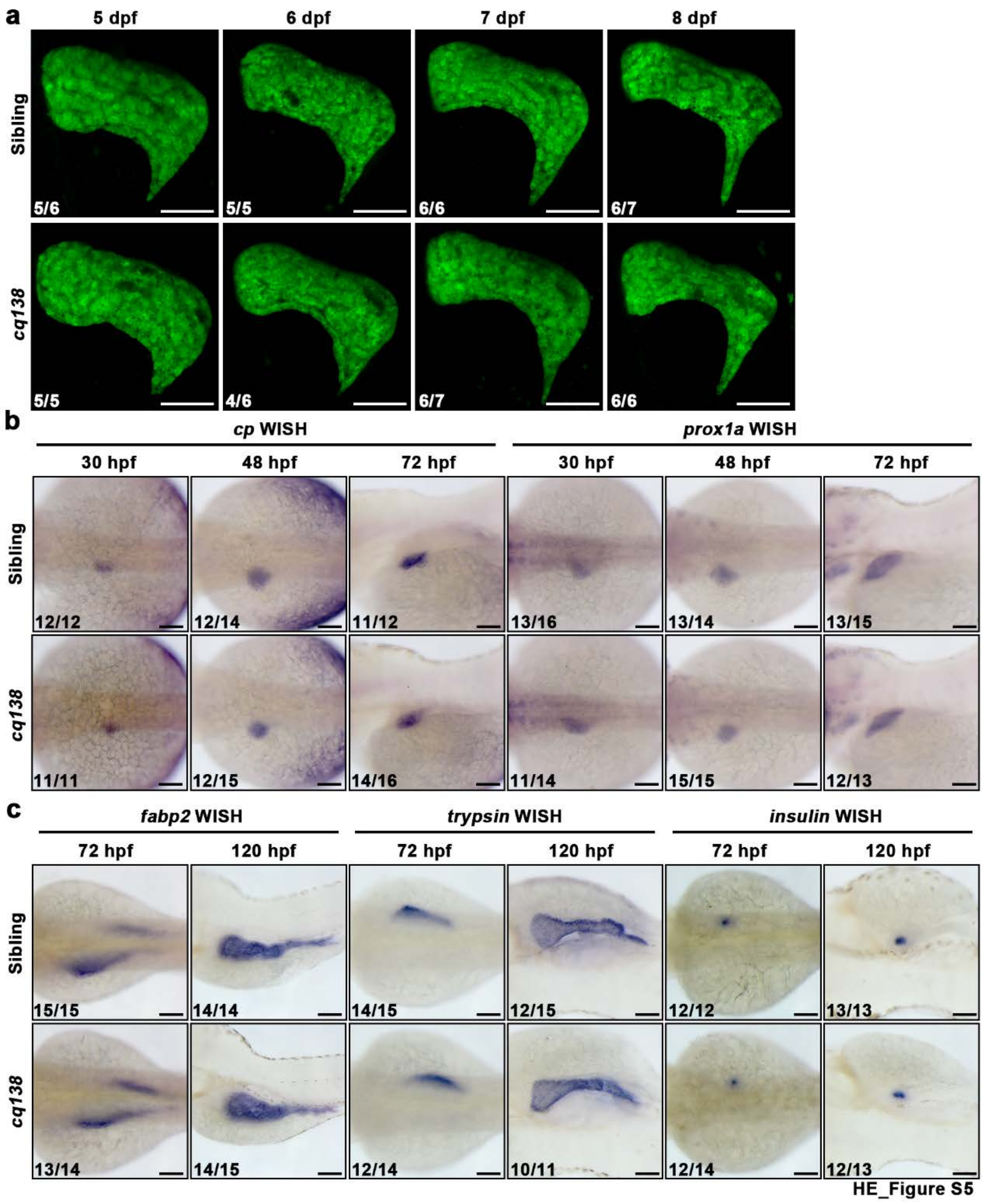

Supplementary Figure 5. The *cq138* mutant exhibits normal development of digestive organs.

a Confocal projection images showing liver development in sibling and *cq138* mutant from 5 dpf to 8 dpf. **b** WISH images showing the expressions of *cp* and *prox1a* at 30 hpf (dorsal view), 48 hpf (dorsal view), and 72 hpf (lateral view) in sibling and *cq138* larvae. **c** WISH images showing the expressions of

fabp2, *trypsin*, and *insulin* at 72 hpf (dorsal view) and 120 hpf (lateral view) in sibling and *cq138* larvae. Numbers indicate the proportion of larvae exhibiting the expression shown. Scale bars, 100 μm. Abbreviations: WISH, whole-mount *in situ* hybridization, dpf, days post-fertilization.

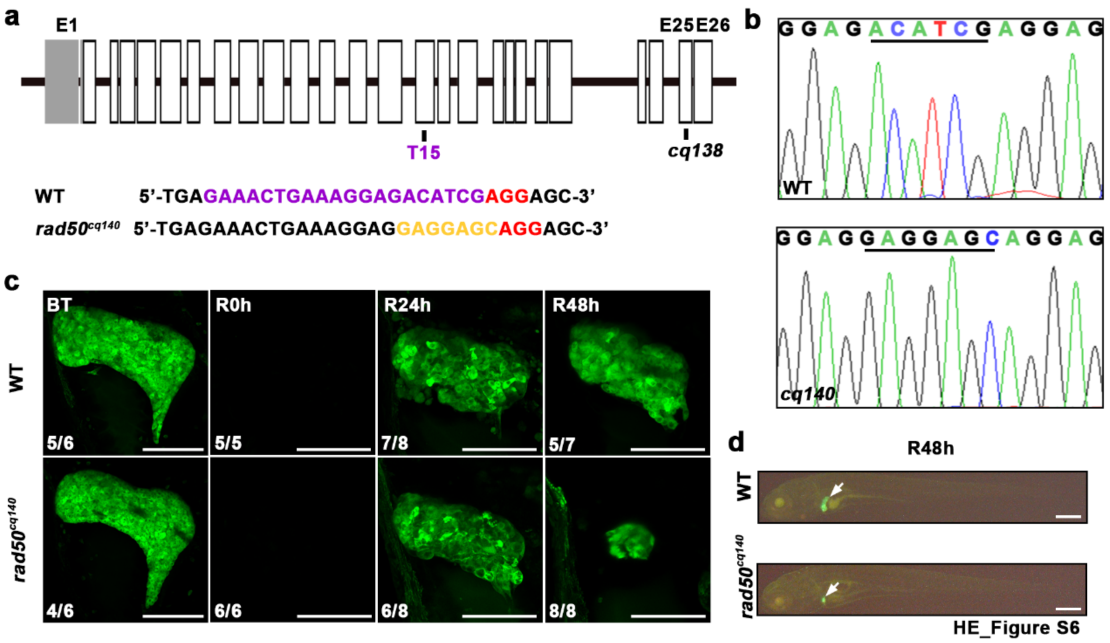

Supplementary Figure 6. Rad50 is required for liver regeneration in zebrafish.

a Diagram showing the genomic structure of *rad50* (top panel) and the alignment of gRNA target sequence in *rad50* exon 15 (purple) (bottom panel). **b** Sequencing result displaying the mutation site in *rad50*. **c** Confocal projection images showing the regenerating livers of wild-type and *rad50*^{cq140} mutant from BT to R48h under the *Tg(lfabp:DenNTR)* background. **d** Epifluorescence images showing the regenerating livers (arrows) of wild-type and *rad50*^{cq140} larvae at R48h. Numbers indicate the proportion of larvae exhibiting the expression shown. Scale bars, 100 μm (**c**) and 300 μm (**d**). Abbreviations: BT, before Mtz treatment; R, regeneration time after the withdrawal of Mtz; WT, wild-type.

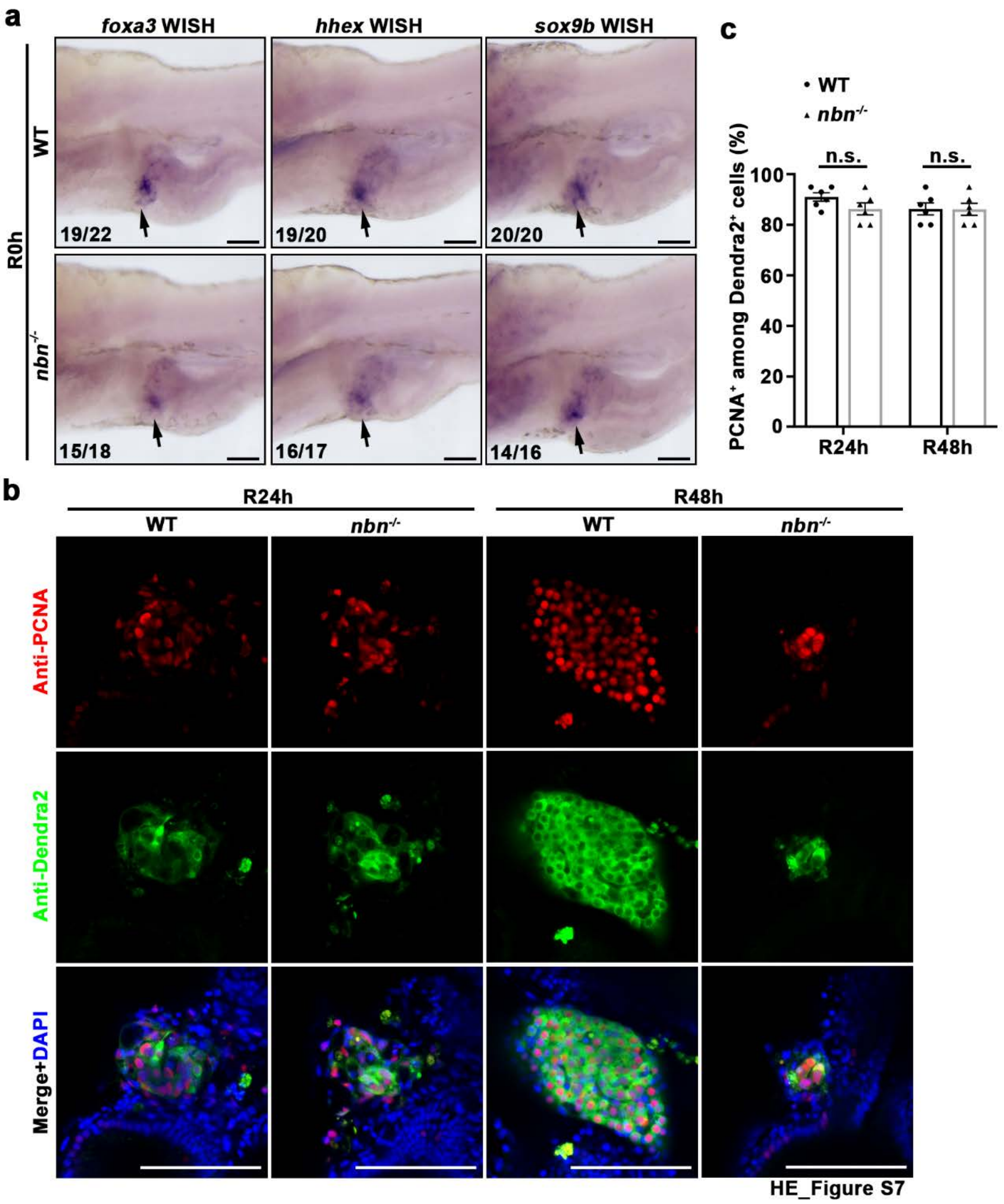

Supplementary Figure 7. Dedifferentiation and cell proliferation are unaffected in *nbn* mutant.

a WISH images showing the expressions of *foxa3*, *hhex*, and *sox9b* in regenerating livers at R0h in wild-type and *nbn* mutant. **b** Single-optical section images showing PCNA (red), Dendra2 (green), and DAPI (blue) staining at R24h and R48h in wild-type and *nbn* mutant. **c** Quantification of the

percentage of $PCNA^+$ among $Dendra2^+$ cells, n = 6 larvae per group. Numbers indicate the proportion of larvae exhibiting the phenotype shown. Scale bars, 100 μm; error bars, ±SEM. Abbreviations: R, regeneration time after the withdrawal of Mtz; WT, wild-type; DAPI, 4’, 6-diamidino-2-phenylindole; n.s., no significance.

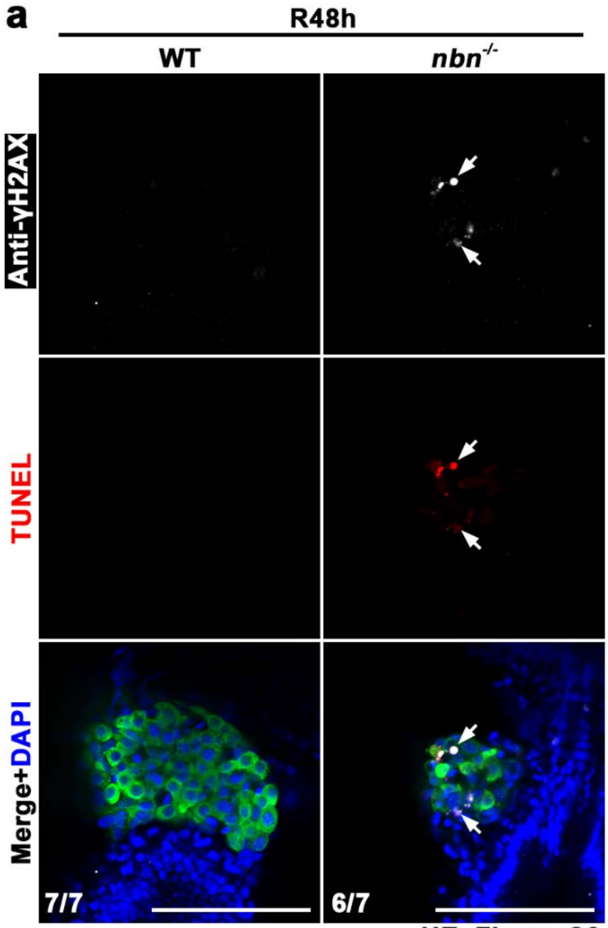

HE_Figure S8

Supplementary Figure 8. The γH2AX positive cells in *nbn* mutant undergo apoptosis.

a Single-optical section images showing γH2AX (white), TUNEL (red), Dendra2 (green), and DAPI (blue) staining at R48h in wild-type and *nbn* mutant. Note that the γH2AX-positive cells are TUNEL-positive in *nbn* mutant liver (arrows). Numbers indicate the proportion of larvae exhibiting the phenotype shown. Scale bars, 100 μm. Abbreviations: WT, wild-type; DAPI, 4’, 6-diamidino-2-phenylindole.

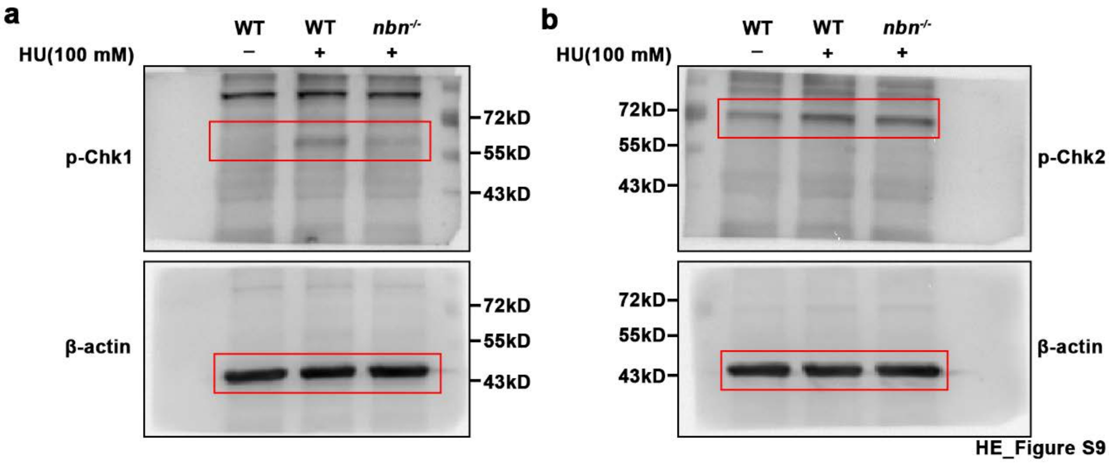

Supplementary Figure 9. The phosphorylation levels of Chk-1 and Chk-2.

a Un-cropped image of p-Chk1. β-actin is used as a loading control. **b** Un-cropped image of p-Chk2. β-actin is used as a loading control. All gels and blots derive from the same experiment and were processed in parallel. Abbreviations: WT, wild-type; HU, hydroxyurea.

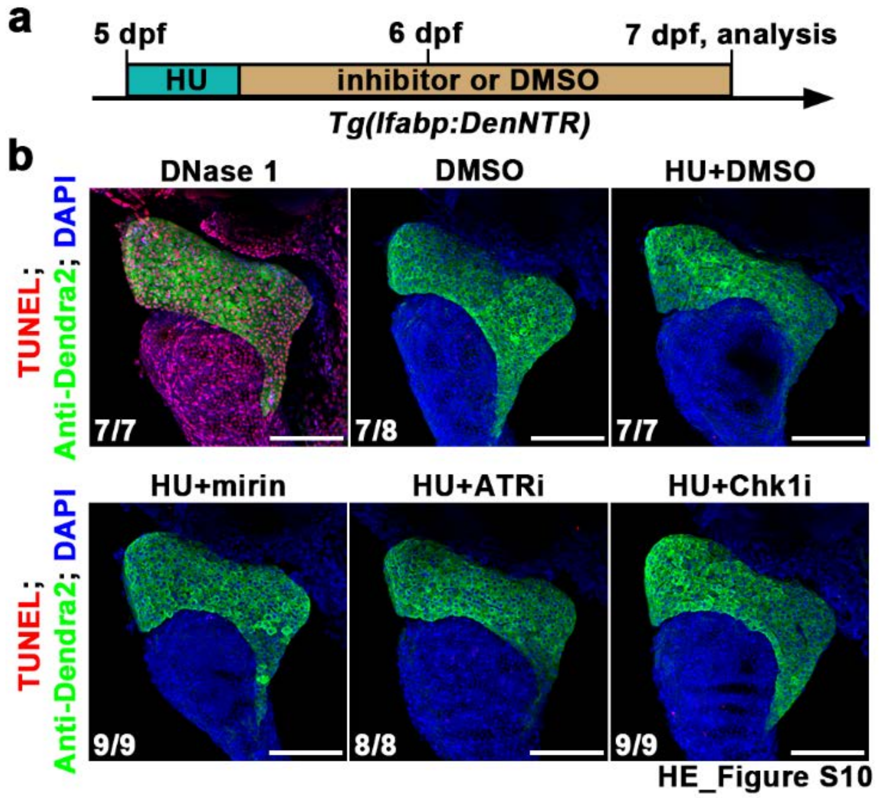

Supplementary Figure 10. The inhibitors do not cause cell apoptosis in hepatocytes.

a Schematic illustration of HU and inhibitor treatment and analysis at 7 dpf. **b** Confocal z-stack projection images showing the label of TUNEL (red), Dendra2 (green), and DAPI (blue) in livers. Note that HU, mirin, ATRi, or Chk1i treatment shows no apoptosis in liver. Numbers indicate the proportion of larvae exhibiting the expression shown. Scale bars, 100 μm. Abbreviations: DAPI, 4’, 6-diamidino-2-phenylindole; HU, hydroxyurea.

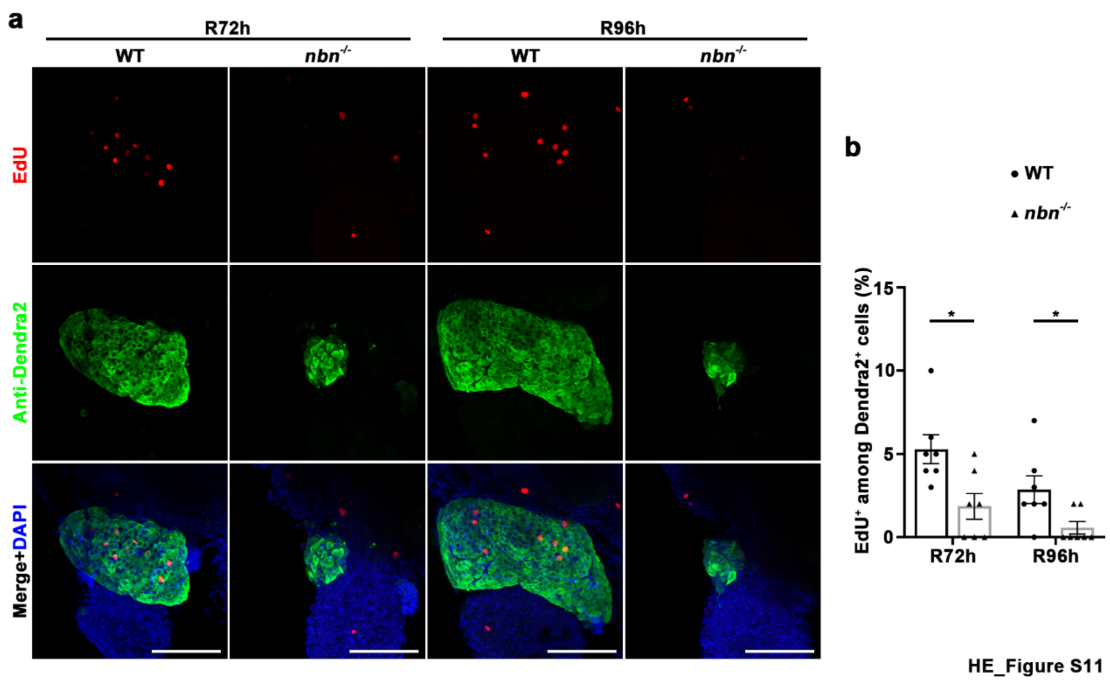

Supplementary Figure 11. The *nbn* mutant exhibits decreased cell proliferation in the later stage of liver regeneration.

a Confocal z-stack projection images showing the staining of EdU (red), Dendra2 (green), and DAPI (blue) in regenerating livers at R72h and R96h in wild-type and *nbn* mutant. Note that number of EdU-positive cells are reduced in *nbn* mutant livers compared to wild-type. **b** Quantification of the percentage of EdU^+ cells among $Dendra2^+$ cells, n = 7 larvae per group. Asterisks indicate statistical significance: *$P < 0.05$ using t-tests analysis when compared to control. Scale bars, 100 μm; error bars, ±SEM. Abbreviations: R, regeneration time after the withdrawal of Mtz; WT, wild-type; DAPI, 4’, 6-diamidino-2-phenylindole; n.s., no significance.

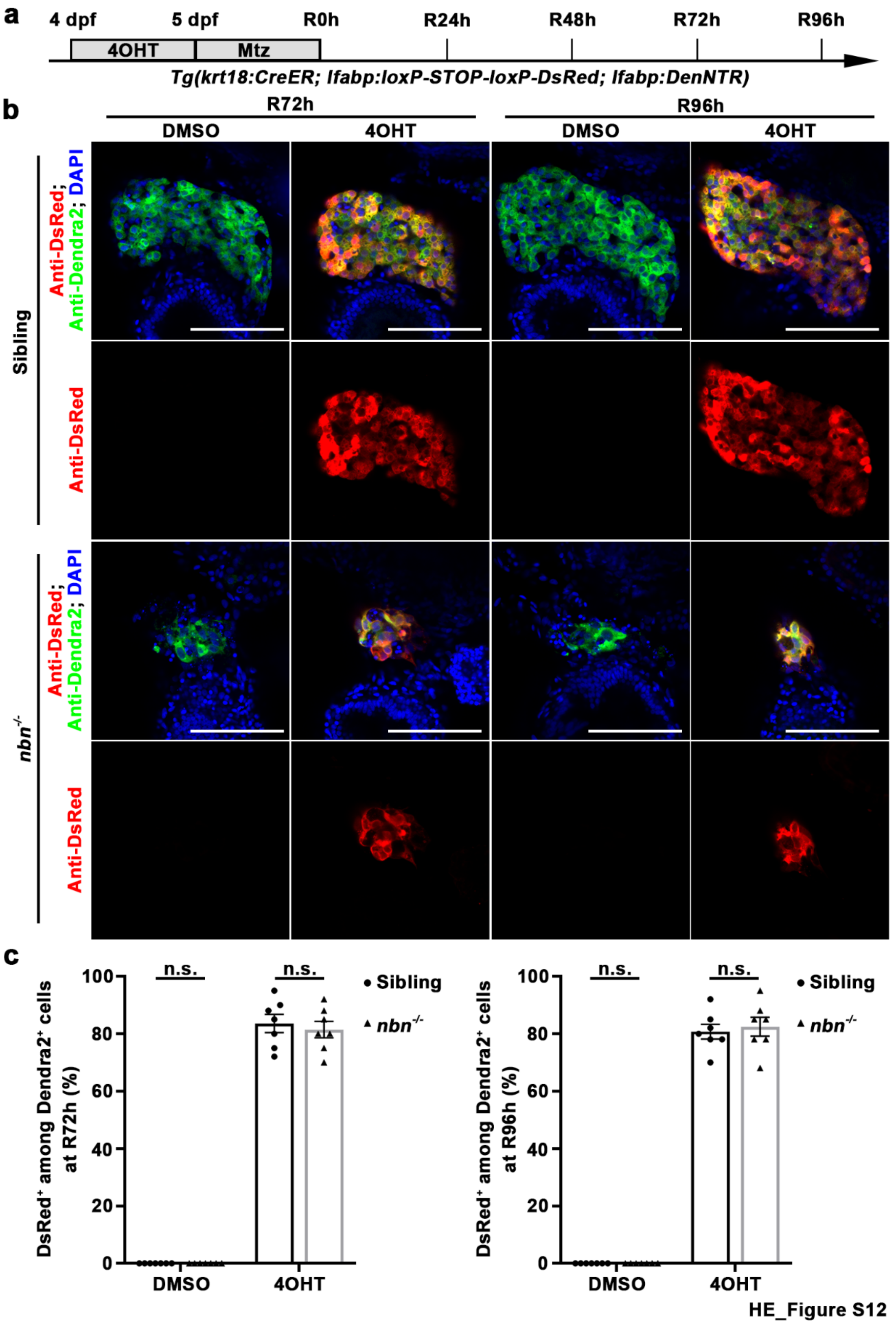

Supplementary Figure 12. Nascent regenerating hepatocytes are derived from BECs in *lvv* mutant during liver regeneration.

a Schematic illustration of the 4OHT or DMSO treatment and analysis at R72h and R96h after Mtz treatment in *Tg(krt18:CreER; lfabp:loxP-STOP-loxP-DsRed; lfabp:DenNTR)* transgenic line. **b** Single optical section images showing the expressions of DsRed and Dendra2 in regenerating livers at R48h. **c** Quantification of the percentage of DsRed$^+$ cells among Dendra2$^+$ cells, n = 7 larvae per group. Scale bars, 100 μm; error bars, ±SEM. Abbreviations: R, regeneration time after the withdrawal of Mtz; DAPI, 4’, 6-diamidino-2-phenylindole; n.s., no significance.

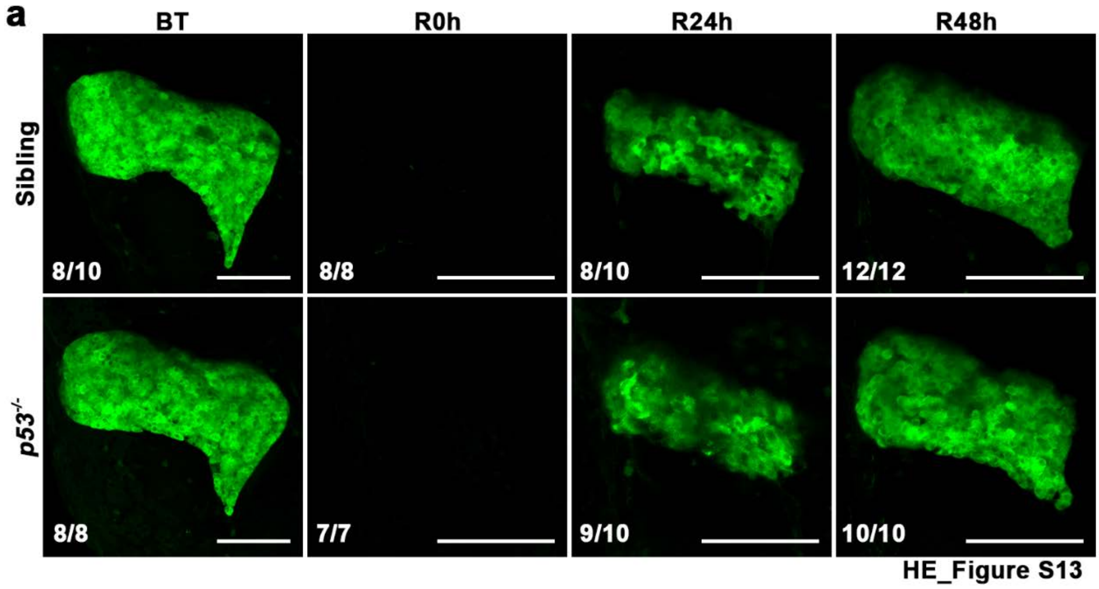

Supplementary Figure 13. Liver regeneration is unaffected in *p53* mutant.

a Confocal projection images showing the regenerating livers of sibling and *p53* mutant from BT to R48h under the *Tg(lfabp:DenNTR)* background. Note that liver regeneration is normal in *p53* mutant. Numbers indicate the proportion of larvae exhibiting the expression shown. Scale bars, 100 μm. Abbreviations: BT, before Mtz treatment; R, regeneration time after the withdrawal of Mtz.

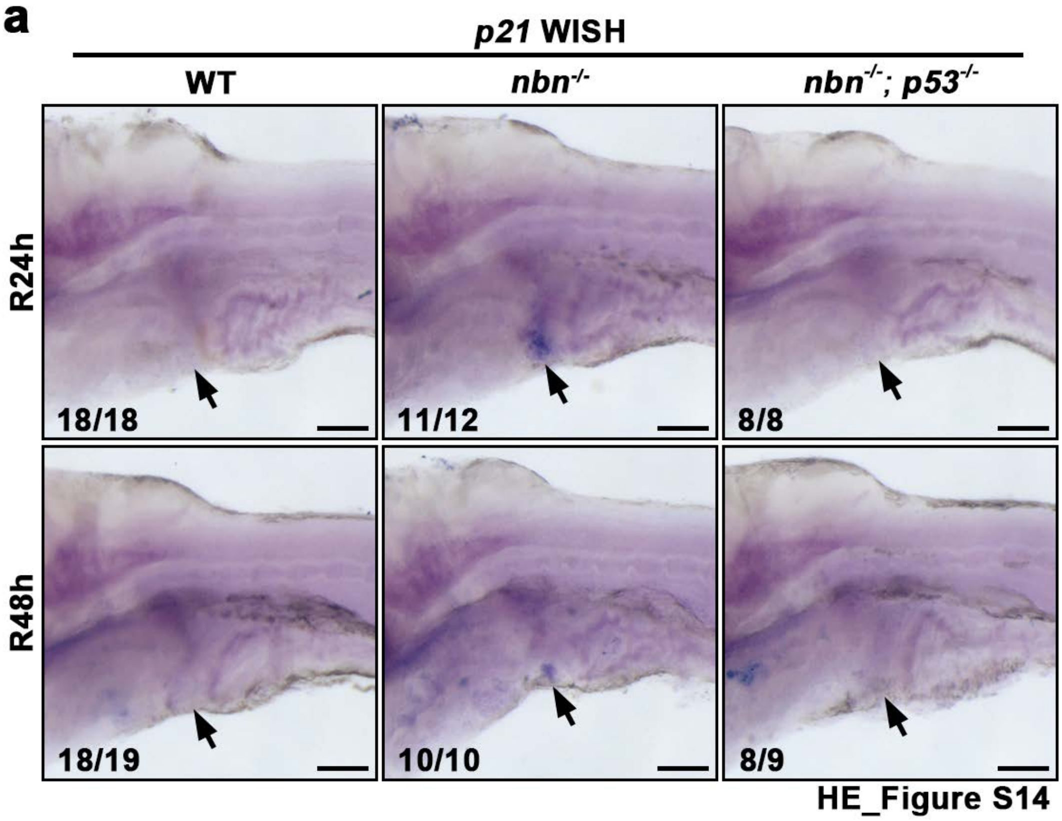

Supplementary Figure 14. The expression of *p21* is p53-dependent in *nbn* mutant.

a WISH result showing the expression of *p21* in wild-type, *nbn*$^{-/-}$, and *nbn*$^{-/-}$ *p53*$^{-/-}$ double mutant regenerating livers at R24h and R48h. Numbers indicate the proportion of larvae exhibiting the expression shown. Arrows indicate the liver region. Scale bars, 100 µm. Abbreviations: R, regeneration time after the withdrawal of Mtz; WT, wild-type; WISH, whole-mount *in situ* hybridization.

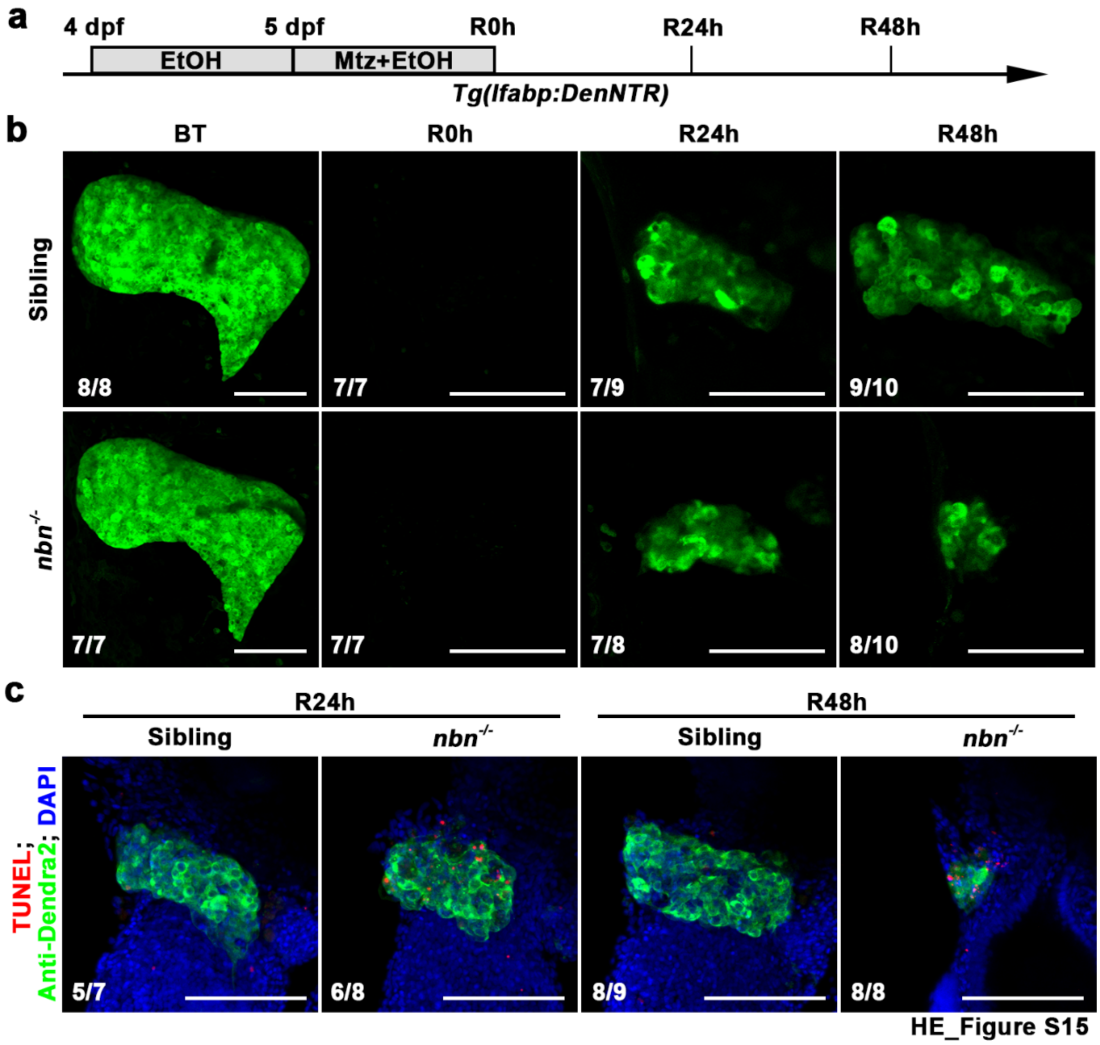

Supplementary Figure 15. Liver regeneration of *nbn* mutant is defective in the fibrotic liver injury model.

a Schematic illustration of the EtOH and Mtz treatment and analysis at R24h and R48h under the *Tg(lfabp:DenNTR)* background. **b** Confocal projection images showing the regenerating livers of sibling and *nbn* mutant from BT to R48h. **c** Confocal z-stack projection images showing the staining of TUNEL (red), Dendra2 (green), and DAPI (blue) in livers. Note that the number of TUNEL-positive cells is increased in *nbn* mutant livers. Numbers indicate the proportion of larvae exhibiting the expression shown. Scale bars, 100 μm.

Abbreviations: R, regeneration time after the withdrawal of Mtz; DAPI, 4’, 6-diamidino-2-phenylindole.

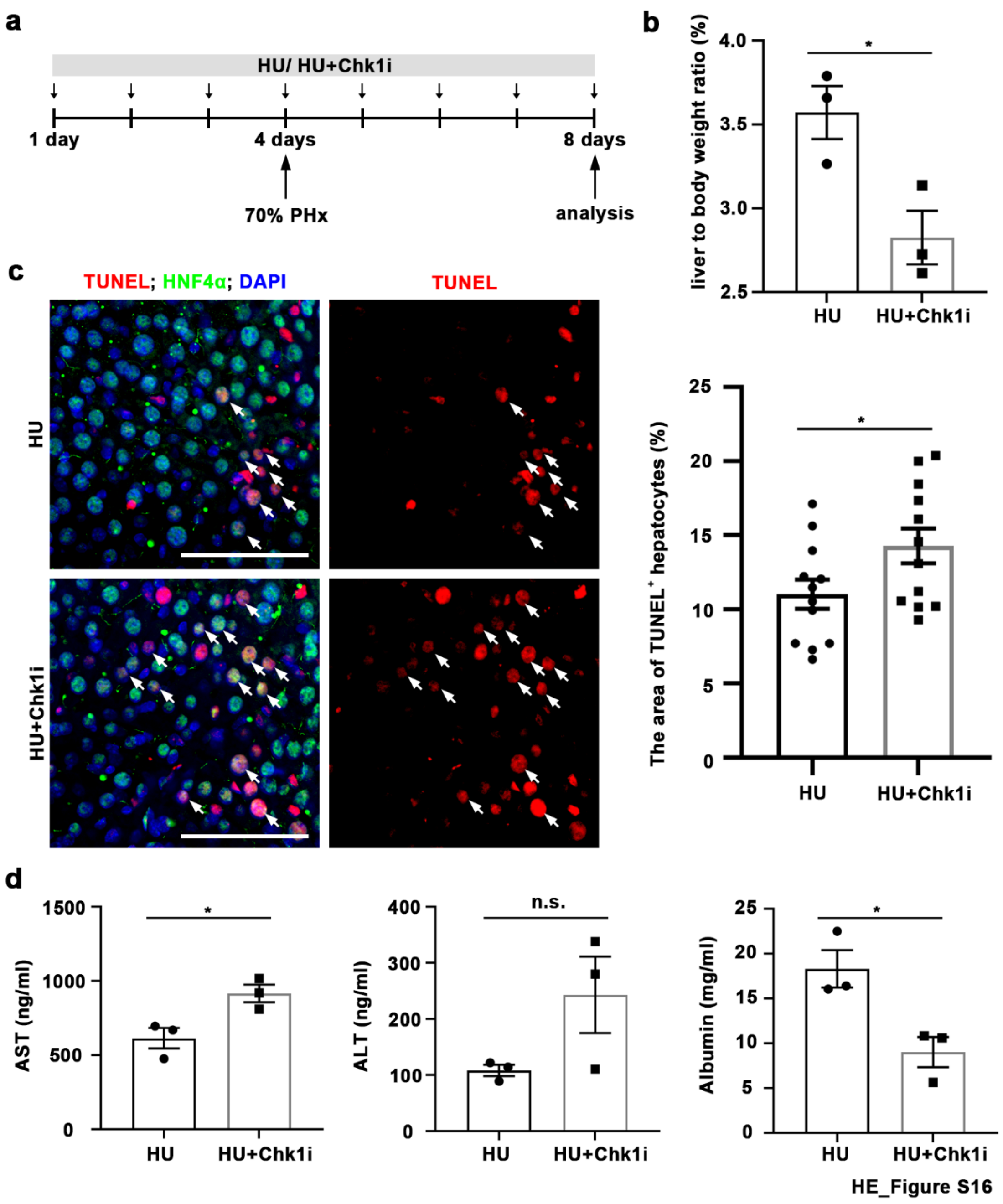

Supplementary Figure 16. Chk1 inhibition impairs liver regeneration after partial hepatectomy in mice.

a Schematic illustration of the period of HU and CHK1 inhibitor treatment (once a day). **b** Quantification of the liver-to-body weight ratio (n = 3) in HU and HU plus CHK1 inhibition groups. **c** Single optical section images showing the TUNEL and HNF4α staining in liver sections and their quantifications (n =

3 biological replicates for each group). Note that the Hnf4a+ $TUNEL^+$ cells are increased after CHK1 inhibitor treatment (arrows). **d** Quantification of the levels of AST, ALT, and Albumin in serum (n = 3) in HU and HU plus CHK1 inhibition groups. Asterisks indicate statistical significance: *P < 0.05 using t-tests analysis when compared to control. Scale bars, 100 μm; error bars, ±SEM. Abbreviations: Chk1i, CHK1 inhibitor CHIR-124; DAPI, 4',6-diamidino-2-phenylindole; HU, hydroxyurea; PHx, partial hepatectomy; n.s., no significance.

Supplementary Tables

Supplementary Table 1. List of Fish strains used in this study.

Fish strain	Function	Source
Tg(lfabp:Dendra2-NTR)^{cq1}	Hepatocyte-specific ablation	(He et al., 2014)[1]
Tg(Tp1:Tomato)^{cq109}	Tomato fluorescent protein under the control of an element containing 12 RBP-Jκ binding sites marks BECs	(zhang et al., 2021)[2]
Tg(krt18:CreER^{cq74}*; βactin:loxP-DsRed-loxP-GFP*^{s928}*)*	Cre/loxP lineage tracing system induced by CreER and 4OHT in BECs	(He et al., 2019)[3], (Kikuchi et al., 2010)[4]
Tg(krt18:CreER^{cq74}*; lfabp:loxP-STOP-loxP-DsRed2*^{cq4}*)*	Cre/loxP lineage tracing system induced by CreER and 4OHT in hepatocytes	(He et al., 2019)[3], (He et al., 2014)[1]
Tg(hsp70l:nbn-p2A-mCherry)^{cq141}	Nbn overexpression via heat shock	This paper
Tg(hsp70l:Myc-nbn^{WT}*)*^{cq142}	Myc-tagged wild-type Nbn overexpression via heat shock	This paper
Tg(hsp70l:Myc-nbn^{cq137}*)*^{cq143}	Myc-tagged mutated Nbn overexpression via heat shock	This paper
p53^{M214K}	*p53* mutant	(Berghmans et al., 2005)[5]
nbn^{cq137}	ENU screen mutant specifically affects liver regeneration	This paper
rad50^{cq138}	ENU screen mutant specifically affects liver regeneration	This paper
nbn^{cq139}	*nbn* mutant by CRISPR/Cas9	This paper
rad50^{cq140}	*rad50* mutant by CRISPR/Cas9	This paper

Supplementary Table 2. List of primers used in this study.

**Name**	**Primer Sequence (5'-3')**
**Primers of SSLP markers**	
Z6021_F	CTGCCCCACAGGAACATC
Z6021_R	TTGCCTTGAACATTTTCATCC
Z3633_F	GCATCCTTTTGTTTGCATGTGC
Z3633_R	TGAGATTTGCGCTTGTGGGA
L16m1_F	GCAGCAGAATACAATACAGATC
L16m1_R	CTTAAGCTGCATTTGAAATGCTG
L16m2_F	CTGTTCAGTGCTTCAATGGATC
L16m2_R	CCAAAAGTGTATCATGGCGAAG
**Primers for genomic DNA sequencing**	
nbn^{cq137}_F	CACATTTCAGGATGGTTACAAG
nbn^{cq137}_R	CTAGAACTAGACTTGCCTTTTC
nbn-T11_F	CAATGTATAGTGGCACAAAGTG
nbn-T11_R	GTCAGTCCTGACTCAAGACG
nbn-T15_F	CTGCACAATTCTGCTTCTCTG
nbn-T15_R	CAGTCAGATCCATGCTCTTTATC
rad50^{cq138}_F	ACAGTACCAAACACGTACTTACAA
rad50^{cq138}_R	GCTCCACGAAATCCTCGTCA
rad50-T15_F	ATGACAGGCAGTCCTTAGTGG
rad50-T15_R	AGACCTGAAAGCGATCCATGAG
**Primers for probe synthesis**	
*nbn*_F	GAGCTCTAAACAGATGAAGCG
*nbn*_R	ATTGTAATACGACTCACTATAGGGGCTGGATGTCATCTTCATTATC
*p53*_F	AAGAACAGCCTCAGCCATCC
*p53*_R	ATTGTAATACGACTCACTATAGGGTCCATTCAGCACCAAGCTGT
*p21*_F	CAGAGGCAGCAGAAAAACTCC
*p21*_R	ATTGTAATACGACTCACTATAGGGTCGTCTCTGGTTCCTGAAGGT
*mdm2*_F	TAACCGAGGCAGACTACTGG
*mdm2*_R	ATTGTAATACGACTCACTATAGGGGCTCGGGGAACTCTTTTTCA
*foxa3*_F	CGAGCGCCATGAACTCAGTG
*foxa3*_R	ATTGTAATACGACTCACTATAGGGGTGCCCTTGGTGCTGCTGC
*hhex*_F	CGAACTCCTCTTTCACCAGCC

*hhex*_R	ATTGTAATACGACTCACTATAGGGCATAGGGTGAACTG ATGCTCG
*sox9b*_F	GGGCTGAAGATGAGTGTGTC
*sox9b*_R	ATTGTAATACGACTCACTATAGGGGATGACATCACTGC TCAGCT
*cp*_F	GATCTGAGACAGACATCCAC
*cp*_R	ATTGTAATACGACTCACTATAGGGGGCTGACGGTGTC GACATG
*gc*_F	CCTCCAAGTCATTGGAATTG
*gc*_R	ATTGTAATACGACTCACTATAGGGCGGAATGGGTACGA CTGGA
*bhmt*_F	GACCTGCTGATCGCTGAGTAC
*bhmt*_R	ATTGTAATACGACTCACTATAGGGGTCTCAGTGTTTAG CGTCCG
*prox1a*_F	GTCCAACGTGCTTCGCAAGC
*prox1a*_R	ATTGTAATACGACTCACTATAGGGGCATGGAGGTTGGT CCCTGG
*fabp2*_F	GCCCTTGTCATCATCATGACC
*fabp2*_R	ATTGTAATACGACTCACTATAGGGGCCCTTGGAGTGCA GATAA
*insulin*_F	GGTCGTGTCCAGTGTAAGCA
*insulin*_R	ATTGTAATACGACTCACTATAGGGCAGGTGTTTCTGGC ATCGG
**Primers for full length cloning**	
*nbn*_F	CGCCAGTTCGTCGGTATCTT
*nbn*_R	TCGTTTCTTTGCAGGCCTAG
**Primers used in site directed mutagenesis mutants**	
nbn^{mut}_F	caaccaataaaccctAatcccttggtcagga
nbn^{mut}_R	tcctgaccaagggatTagggtttattggttg
**CRISPR/Cas9 targeting site sequence**	
nbn-T11	GAACTGGCACTGCTTTCACGCGG
nbn-T15	GGAGAAGCTGAACGAACGAGAGG
rad50-T15	GAAACTGAAAGGAGACATCGAGG

Underlined are T7 primer sequences.

Supplementary References

1. He, J., Lu, H., Zou, Q. & Luo, L. Regeneration of liver after extreme hepatocyte loss occurs mainly via biliary transdifferentiation in zebrafish. *Gastroenterology* **146**, 789-800 (2014).
2. Zhang, W. *et al.* Contributions of biliary epithelial cells to hepatocyte homeostasis and regeneration in zebrafish. *iScience* **24**, 102142 (2021).
3. He, J. *et al.* Mammalian Target of Rapamycin Complex 1 Signaling Is Required for the Dedifferentiation From Biliary Cell to Bipotential Progenitor Cell in Zebrafish Liver Regeneration. *Hepatology* **70**, 2092-2106 (2019).
4. Kikuchi, K. *et al.* Primary contribution to zebrafish heart regeneration by gata4+ cardiomyocytes. *Nature* **464**, 601-605 (2010).
5. Berghmans, S. *et al.* tp53 mutant zebrafish develop malignant peripheral nerve sheath tumors. *Proc. Natl. Acad. Sci. USA* **102**, 407-412 (2005).
